# 1-Butyl-3-methylimidazolium-Based Ionic Liquid in Biomass Fractionation—Green Solvent or Active Reagent Toward Lignin Compounds?

**DOI:** 10.3390/ijms252312623

**Published:** 2024-11-25

**Authors:** Artyom V. Belesov, Dmitrii M. Mazur, Anna V. Faleva, Ilya S. Varsegov, Ilya I. Pikovskoi, Nikolay V. Ulyanovskii, Dmitry S. Kosyakov

**Affiliations:** 1 Core Facility Center ‘Arktika’, Northern (Arctic) Federal University, Arkhangelsk 163002, Russia; a.belesov@narfu.ru (A.V.B.); a.bezumova@narfu.ru (A.V.F.); i.varsegov@narfu.ru (I.S.V.); i.pikovskoj@narfu.ru (I.I.P.); n.ulyanovsky@narfu.ru (N.V.U.); 2 Department of Materials Science, MSU-BIT University, Shenzhen 517182, China; 3 Department of Organic Chemistry, Lomonosov Moscow State University, Moscow 119991, Russia

**Keywords:** lignin, ionic liquids, 1-butyl-3-methylimidazolium

## Abstract

N,N′-Dialkylimidazolium-based ionic liquids are capable of completely dissolving lignocellulosic biomass at elevated temperatures and are considered as promising green solvents for future biorefining technologies. However, the obtained ionic liquid lignin preparations may contain up to several percent nitrogen. This indicates strong interactions between the biopolymer and the IL cation, the nature of which has not yet been clarified. The present study investigates mechanisms and pathways of the formation of nitrogen-containing lignin compounds. To achieve this goal, eight monomeric lignin-related phenols bearing different functional groups (ketone, aldehyde, hydroxyl, carbon–carbon double bonds) were treated with 1-butyl-3-methylimidazolium acetate (BmimOAc) under typical conditions of IL-assisted lignocellulose fractionation (80–150 °C). A number of the resulting products were tentatively identified, for all the studied model compounds, by two-dimensional NMR spectroscopy and high-performance liquid chromatography—high-resolution mass spectrometry. They all possess covalently bonded Bmim residues and occur through the nucleophilic addition of an N-heterocyclic carbene (deprotonated Bmim cation) to electron-deficient groups. The reactivity of lignin functional groups in their interaction with Bmim is greatly affected by the temperature and dissolved oxygen. IL’s thermal degradation products act as additional reactive species toward lignin, further complicating the range of products formed. The obtained results made it possible to answer the question posed in this article’s title and to assert that N,N′-dialkylimidazolium-based ILs act as active reagents with respect to lignin during the dissolution of lignocellulose.

## 1. Introduction

The fractionation of lignocellulose using ionic liquids (ILs) is considered a promising environmentally friendly approach to the complete valorization of renewable plant biomass (biorefining) that allows for obtaining a wide range of high value-added chemicals and materials [1,2,3,4,5,6]. Due to their ionic nature, ILs possess unique properties such as a low vapor pressure, inflammability, thermal stability within a limited temperature range, and the ability to be regenerated and reused in technological processes under certain conditions [7,8,9,10,11]. Many properties of ILs, including thermal stability, may be varied widely by replacing the cation or anion in their composition [11] or adding molecular solvents [12]. The disadvantages of ionic liquids include their high cost, the significant toxicity of some common ions, including the widely used imidazolium derivatives, and their reduced biodegradability [13]. They result in the partial displacement of ILs by more available and safer deep eutectic solvents [14].

One of the key features of many ILs (primarily based on various alkylammonium and alkylimidazolium cations [15]) is a high dissolving power for both lignin and polysaccharides, making them the only single-component liquids capable of dissolving plant biomass entirely [15,16]. This especially applies to the ILs based on the N,N′-dialkylimidazolium (typically, 1-butyl-3-methylimidazolium, Bmim, and 1-ethyl-3-methylimidazolium, Emim) cation and various anions (acetate, chloride, methyl sulfate) that are considered promising media for future biorefining technologies [17,18]. The dissolution of biomass is achieved at elevated temperatures (up to 150 °C) and its individual components (lignin, cellulose, hemicelluloses) can be separated by selective anti-solvent precipitation or other methods. While the resulting polysaccharide fractions find common applications, efficient methods for the valorization of ionic liquid lignin (ILL) have not yet been developed. This is due to the complexity of the biopolymer’s structure, being built from *p*-hydroxyphenyl, guaiacyl, and syringyl-type phenylpropane units linked with various bonds (alkyl-aryl β-O-4 and α-O-4, aryl-aryl 5-5, alkyl-alkyl β-β), and the poorly understood interactions that occur during lignin dissolution in ILs.

It is well-known that ILs can interact with lignin, causing its chemical transformation [19]. At elevated temperatures, the action of ILs primarily leads to the destruction of the most abundant and labile β-O-4 ether bonds, leading to the partial depolymerization of lignin [20,21]. At the same time, condensation can also occur through the formation of α-5 and β-5 carbon–carbon bonds [22]. The most intriguing fact is the presence of nitrogen in the elemental composition of lignin preparations obtained using Bmim- and Emim-based ILs. The average content of nitrogen varies widely (0.5–10%) depending on the IL’s anion and the applied biomass fractionation conditions [23]. Some previous studies have explained this observation by suggesting the incomplete removal of ILs from lignin due to the action of strong intermolecular forces between ILs and some functional groups of biomacromolecules [24,25], considering ILs as neutral solvents not capable of covalent bonding to lignin substances. However, more recent research has shown that some alkylammonium-based ILs cause the amination of lignin [23]. Studying the properties of an IL lignin preparation isolated from wood with Bmim acetate, Ladesov et al. [26] suggested the possibility of covalent interactions between the Bmim cation and lignin. Later, a large number of N-containing oligomers was detected in spruce IL (BmimOAc and BmimMeSO_4_) lignins by atmospheric pressure photoionization high-resolution mass spectrometry (APPI-HRMS) and ^13^C NMR spectroscopy [27]. Although their exact structure remained unclear, the inclusion of Bmim cation residue in it was established. Similar results were obtained by MALDI mass spectrometry, which allowed for the detection of hundreds of N-containing compounds with molecular weights up to 1500 Da [28]. Their tandem mass spectra showed the elimination of Bmim (*m*/*z* 139), indicating covalent binding of the IL cation to lignin oligomers. It was suggested that the main mechanism of such interactions involves the formation of N-heterocyclic carbene (NHC) [29] as a reactive intermediate possessing both nucleophilic and basic properties. This process proceeds through the deprotonation of a dialkylimidazolium cation and is catalyzed by bases, which can be an IL’s anions. The NHC can react with non-enolizable carbonyl substrates to form the so-called “Breslow” intermediate [30,31], which in turn initiates condensation processes such as benzoin condensation. This reaction occurs predominantly at a temperature of ≥120 °C [29] in the absence of aldehyde structures in the reaction mixture [31]. The latter may form adducts with Bmim via nucleophilic attack by NHC. It can be assumed that some other functional groups of lignin may also interact with NHC. Moreover, our recent study [32] demonstrated that, due to the formation of NHC, Bmim-based ILs undergo partial thermal decomposition with the formation of various N-containing products (substituted imidazoles, N-alkylated amines, and amides), which are also capable of interactions with lignin compounds. This may explain the exceptional diversity of IL-modified lignin compounds observed in mass spectra [27,28].

**Figure 1:**
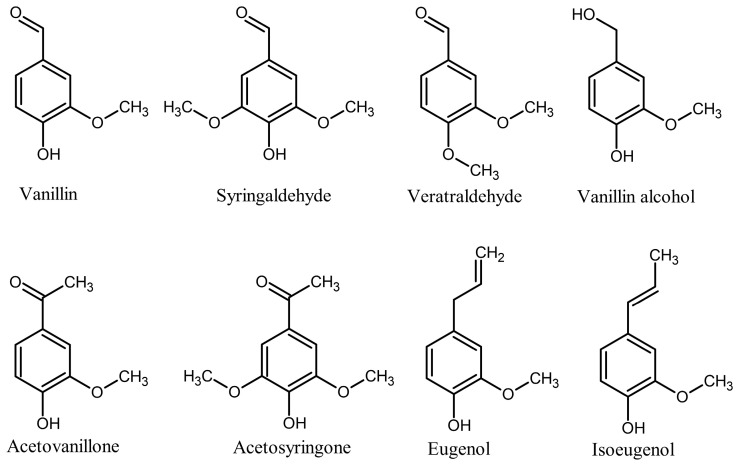
Phenolic substrates mimicking the key structural fragments of lignin macromolecules.

The purpose of this study was to fill the existing gap in understanding of the possibility, potential mechanisms, and pathways of the formation of nitrogen-containing lignin oligomers under typical conditions of IL-assisted lignocellulose treatment (80–150 °C). To achieve this goal, a methodology was used that involved conducting model experiments on the interaction of Bmim acetate with simple monomeric phenolic compounds (Figure 1) mimicking the key structural units of lignin macromolecules and bearing various functional groups (ketone, aldehyde, hydroxyl, and conjugated and non-conjugated carbon-carbon double bonds). The choice of the IL in this study was determined by its high dissolving power toward lignocellulose and the high basicity of its acetate anion, facilitating the formation of NHC and thus reaction products. Their reliable identification was achieved by implementing an advanced analytical methodology involving the combination of two-dimensional NMR and HPLC-HRMS analyses of the reaction mixtures.

## 2. Results and Discussion

### 2.1. Reactions with Aromatic Aldehydes and Alcohols

**Figure 2:**
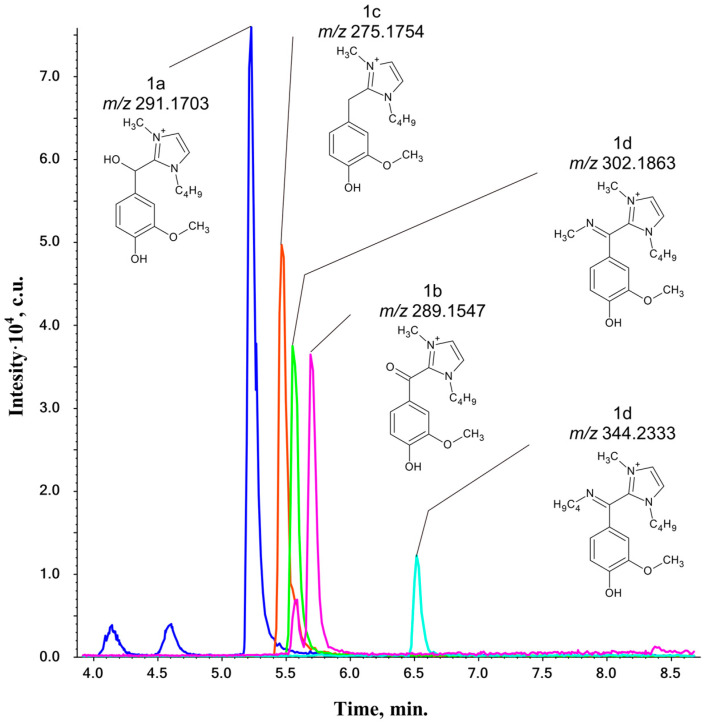
Exact mass-based extracted ion chromatograms (XICs) of reaction products (**1a**–**1d**) of vanillin with BmimOAc (120 °C, 300 min).

As mentioned above, the interactions between the Bmim cation and electron-deficient groups of lignin may result in the formation of various nitrogen-containing compounds, and aldehyde groups are a priority target for IL attack [30,31]. Table 1 summarizes the adducts of BmimOAc with vanillin, syringaldehyde, and veratraldehyde, as detected by HPLC-HRMS (Figure 2).

Accurate mass measurements revealed the presence of two or three nitrogen atoms in the studied elemental compositions. This suggests the addition of both the Bmim cation (N_2_-compounds) and probable thermal degradation products of IL (N_3_-compounds) [32]. The identified products were classified as types from **1a** to **1e** based on the degree of transformation of the substrate.

The obtained tandem mass spectra (Supplementary Figure S1) of the **1a**-type reaction products demonstrate that the most abundant product ions are observed at *m*/*z* 139 ([C_8_H_15_N_2_]^+^) and *m*/*z* 83 ([C_4_H_7_N_2_]^+^) and can be attributed to Bmim and its debutylated derivative, respectively. This suggests that the charge is mainly retained at the Bmim fragment during the fragmentation of the parent molecular ion. The minor peaks in Figure S1 (Supplementary Materials) indicate that the hydroxybenzyl moiety is bonded to the C2-atom of the imidazole cycle, while the rest of the structure comes from the substrate (**1a**, Figure 2). Product **1a** is initially a cation with a charge delocalized in the imidazolium ring. The formation of this product was expected due to the known reaction of NHC with non-enolizable aldehydes, which was described previously [25].

The **1b**-type products differ from those of the **1a** type by two hydrogen atoms, which increases their double bond equivalent (DBE) by 1. Their structure can be proposed by replacing the aliphatic hydroxyl in **1a** with a carbonyl group. The product ions with *m*/*z* 151.0388 and 181.0494 (Supplementary Figure S2) represent [Ar-C=O]^+^ fragments for vanillin and syringaldehyde Bmim adducts, respectively. Additionally, the signal detected at *m*/*z* 109 ([C_5_H_5_N_2_O]^+^) and related to the 2-acyloyl-1-methylimidazole structure supports the idea of a carbonyl group acting as a bridge between the imidazolium cycle and the substituted aromatic ring. The other signals observed in the MS/MS spectra reflect the fragmentation of the Bmim moiety.

The detection of **1c**-type reaction products was unexpected, as they differ from the **1a** type by only one oxygen atom. It is highly unlikely that the methoxy group of veratraldehyde would be substituted with a methyl. Therefore, the **1c**-type compounds were expected to have a hydrogen atom instead of a hydroxyl group in the benzyl position. The main fragmentation processes involve bond cleavages within the imidazolium cycle, resulting in the loss of a methyl radical, butene, or both. However, for structure elucidation, the most important fragment ions are [M-138]^+^ ions, which result from the loss of the Bmim residue (C_8_H_14_N_2_) and likely have a substituted tropylium cation structure, and [C_5_H_7_N_2_]^+^ with *m*/*z* 95 (Supplementary Figure S3). Their detection provides evidence that the Bmim and aromatic fragments are bonded through a methylene group.

Finally, the elemental compositions listed in Table 1 allow us to conclude that products of the **1d** and **1e** types may have resulted from the reaction of **1b** with IL’s degradation products, namely methylamine and *n*-butylamine. The mass spectra presented in Figure S4 (Supplementary Material) reveal the formation of N-alkyl imine derivatives of **1b**-type compounds Ar-C(=NR)-Bmim (R = -CH_3_ or -C_4_H_9_), which are secondary transformation products.

The assumed structures of the detected compounds were confirmed by two-dimensional (2D) ^1^H-^13^C NMR spectroscopy. The HSQC (heteronuclear single quantum coherence) cross-peaks and the correlations between them in the HMBC (heteronuclear multiple bond correlation) spectrum confirm the formation of **1a** and **1c** types, resulting from the interaction of aldehydes with NHC (Supplementary Figures S5–S8). The HSQC spectrum of the **1a**-type compound shows a cross-peak of tertiary a C_10_-OH carbon atom (62.92/6.47 ppm) bonded to the benzene ring Ar_1,3,5_ (109.87/6.47, 127.64/6.47, and 117.48/6.47 ppm, respectively) and Bmim C_11_ (147.55/6.47 ppm), as indicated by the HMBC spectrum. The HSQC spectrum of the **1c** contains a cross-peak of a C_10_-H secondary carbon atom (27.39/4.44 ppm) that is bonded, according to the HMBC spectrum, with the Ar_1,3,5_ benzene ring structure (112.45/4.44, 121.83/4.44, and 119.85/4.44 ppm, respectively) and Bmim C_11_ (145.06/4.44 ppm). However, the cross-peaks observed in the NMR spectra did not allow for confirmation of **1b**- or **1d**/**1e**-type structures. This may be due to their structures containing proton-deficient fragments, specifically a few successive carbon atoms in positions 1, 10, and 11. Correlations between these fragments cannot be determined from the HMBC spectrum.

**Figure 3:**
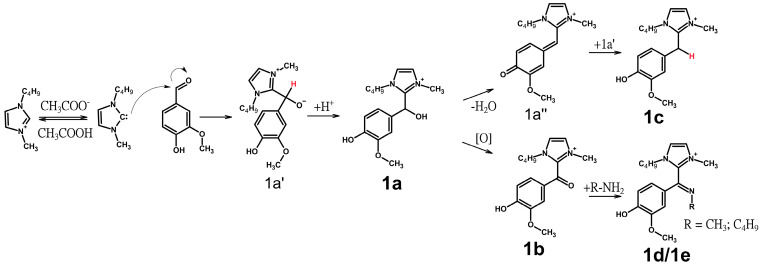
Interactions of aromatic aldehydes (on an example of vanillin) with Bmim acetate.

Summarizing the above, the following scheme (Figure 3) for the formation of the observed reaction products can be proposed:

Since the formation of **1a** was expected based on data from the literature, all further explanations are based on this major product type and its intermediate, **1a′**. To understand the formation of the **1c**-type products, the dehydration of **1a**, resulting in the formation of the highly reactive intermediate **1a″**, can be assumed. Apparently, hydride transfer from **1a′** to **1a″** was the only possible way to achieve complete reduction of the benzyl C-atom. Both the **1b** and **1c**-type products were assumed to be formed in this way. However, it is possible that the **1b**-type compound may also occur as a result of the oxidation of the **1a**-type compounds by air oxygen. The subsequent condensation reaction of the **1b**-type compound with aliphatic amines, such as methylamine and *n*-butylamine, leads to the formation of **1d**- and **1e**-type compounds. In the case of veratraldehyde, the formation of intermediate **1a″** is impossible due to the lack of the free phenolic hydroxyl in its structure, which explains the absence of **1b**-, **1d**-, and **1e**-type products in the reaction mixture. It seems reasonable that the formation of a **1c**-type product from veratraldehyde proceeds trough the protonation of the OH group in the **1a**-type compounds, with further dehydration resulting in the formation of a carbocation. The latter serves as an intermediate, similar to **1a″**, from which the formation of the **1c**-type products occurs subsequent to hydride transfer. Given the absence of the **1b** and **1d/e** types among the detected products, it can be postulated that the source of hydrogen is not **1a′**, but rather one of the degradation products of the IL. However, the precise identity of the compound involved in this process is yet to be determined.

The interaction products found in the reaction mixtures have a similar structure and, already being cations, exhibit similar ionization efficiency under ESI(+) conditions. This enables the sum of areas for all detected products to be taken as 100%, allowing for comparison of the levels of each component in the reaction mixture (Supplementary Table S1). The compound of the **1a**-type appears as the major reaction product in the mixture during the first 10 min of the experiment. Further treatment leads to the accumulation of **1b**-, **1c**-, **1d**-, and **1e**-type products. The temperature of the reaction mixture was found to affect the rate of accumulation and changes in the ratio of the compounds formed. At 80 °C, **1a**- and **1b**-type products were the predominant compounds, while increasing the temperature to 120 °C further promoted the formation of NHC and increased the initial level of **1a** compounds, which is consistent with previous studies [33]. Raising the temperature to 120 °C results in a higher degree of Bmim acetate degradation [31], which in turn stimulates the formation of products of the **1d**- and **1e**-types. An increase to 150 °C promotes the formation of a **1c**-type product, likely by increasing the rate of formation of intermediate **1a″**. For veratraldehyde, the formation of **1e** products is not possible, so only higher levels of **1a** products are observed.

Surprisingly, transformations of vanillin alcohol during the treatment with IL results in the formation of the same products as those formed using vanillin. At the same time, special attention should be drawn to the formation of large quantities of the **1c**-type compound, which is a minor product in the case of the aldehyde. It is believed that **1c** products occur due to the initial dehydration of vanillin alcohol, followed by an attack of NHC. Notably, the **1c** group had the highest levels among all the components of the reaction mixtures at both 80 and 150 °C. Conducting the reaction in an inert argon atmosphere at 150 °C for 6 h leads to the complete absence of **1b**-type products, an order of magnitude decrease in the level of **1a**-type products, and an increase in the level of **1c**-type products compared to the experiment without argon (Supplementary Table S2). This suggests that the formation of **1a**-type products from vanillin alcohol proceeds through an initial oxidation of the latter to an aldehyde and confirms the hypothesis about the role of oxygen in the formation of **1b**-type products from **1a**-type products.

### 2.2. Reactions with Ketones

**Figure 4:**
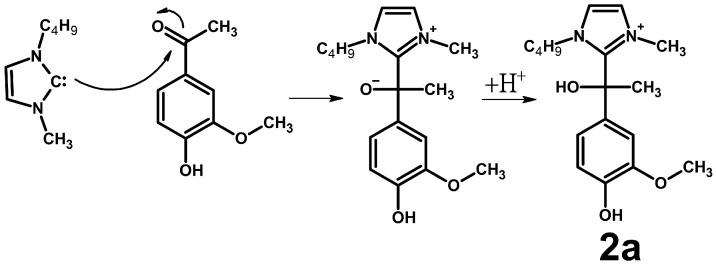
Formation of **2a**-type compound in the reaction of Bmim with acetovanillone.

The discovery of compounds with the elemental compositions [C_17_H_25_N_2_O_3_]^+^ and [C_18_H_27_N_2_O_4_]^+^ among the reaction products of acetovanillone and acetosyringone, respectively, confirms the assumption about the possibility of the nucleophilic addition of NHC to enolizable carbonyl compounds, resulting in the formation of compounds of the **2a** type, which are similar to the **1a** type (Figure 4).

**Figure 5:**
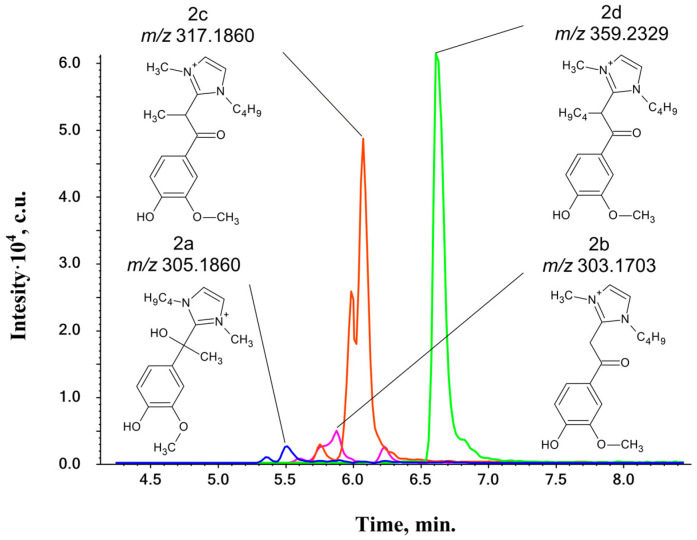
Exact mass-based extracted ion chromatograms (XICs) of reaction products (**2a**–**2d**) of acetovanillone with BmimOAc (150 °C, 300 min).

The HPLC-HRMS analysis of the reaction mixture (Figure 5) shows the formation, along with **2a** products, of three other compounds designated as **2b**–**2d** (Table 2).

Obviously, the formation of **2a** products proceeds similarly to those of the **1a** type. Tandem mass spectra (Supplementary Figure S9) of the **2a**-type products show fragment ions corresponding to a loss of methyl radicals (15 Da), butene, and H_2_O molecules (74 Da), and the elimination of Bmim (*m*/*z* 139, [C_8_H_15_N_2_]^+^) and its fragment (*m*/*z* 83, [C_4_H_7_N_2_]^+^). The observed fragmentation pattern indicates bonding through the C2-atom of the imidazole cycle and the α-carbon atom of the model ketone.

The mass spectra of the **2b**–**2d**-type compounds (Supplementary Figures S10–S12) also contain ions formed during the aforementioned losses of alkyl fragments from the Bmim moiety. However, the most important fragment ion that provides insight into the structure is observed at *m*/*z* 151 (for acetovanillone) and corresponds to the Ar-C=O fragment. This fact suggests that the bonding of Bmim to α-ketones also proceeds through the β-carbon atom of phenol and the C2-atom of the imidazole cycle. Furthermore, the mass spectra of the **2c**- and **2d**-type compounds indicate that additional alkylation of **2b** products has occurred. Based on the observed fragment ions, it is assumed that more alkyl substituents are attached to the same β-carbon atom.

**Figure 6:**
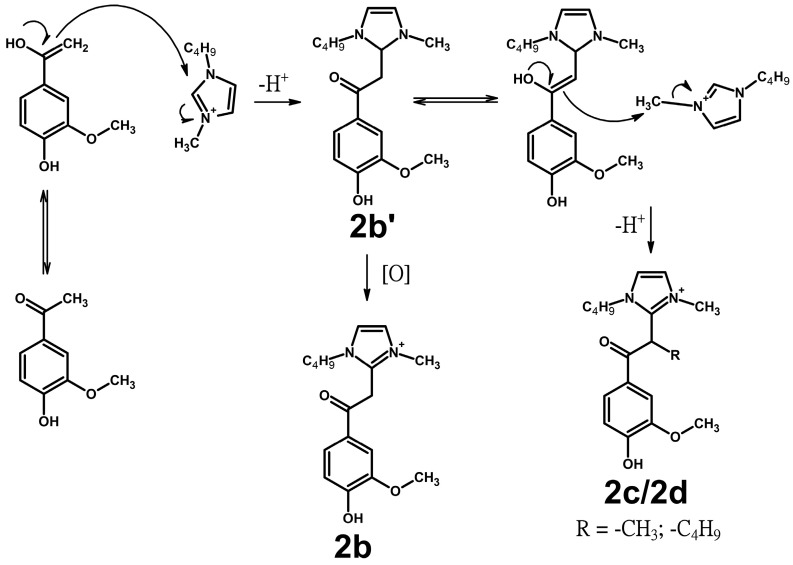
Formation of **2b**-,**c**-,**d**-type compounds in the reaction of Bmim with acetovanillone.

The formation of **2b**–**2d**-type products proceeds at higher temperatures compared to that of **2a** products (Supplementary Table S3), at which noticeable thermal degradation of the IL occurs [32]. The signals of numerous degradation products along with the high-intensity peaks of BmimOAc strongly interfere with the detection of these target compounds in 2D NMR spectra (Supplementary Figures S13–S16), making confirmation of their structure challenging. However, only the C_10_ carbon atom (35–45/4.5–5.5 ppm) has no signal on the HMBC spectra of the reaction mixtures, which may be due to the shielding effect caused by the presence of the conjugated imidazolium cation and the ketone group. Thus, the assumed pathways for the formation of the detected compounds may be presented by the following scheme (Figure 6).

Compounds of the **2b**–**2d** types have not been previously described in the literature, and their formation mechanisms have not been studied. We assume that enolization of the model ketones makes them nucleophilic, leading to an attack on the C_2_-atom of the Bmim cation and production of the product **2b′**. Subsequent oxidation results in the aromatization of the heterocyclic part, returning it to the Bmim fragment in the structure of **2b** products. The enol form of **2b′** may undergo further transformations, such as alkylation by the Bmim cation through the S_N_2 mechanism, to form **2c** and **2d-type** products. According to the literature, dealkylation reactions of Bmim occur at temperatures >120 °C [34,35,36]. This is consistent with the observation that the **2c** and **2d** types only appear in the reaction mixture at these temperatures (Supplementary Table S3). Below 120 °C, only acetovanillone interacts with the IL, preferentially forming the product type **2a**. Increasing the temperature promotes the formation of **2b** and increases in its levels. However, at 150 °C, the thermal decomposition of the Bmim cation dominates, leading to the formation of **2c** and **2d** products.

### 2.3. Reactions with Double C=C Bond

**Figure 7:**
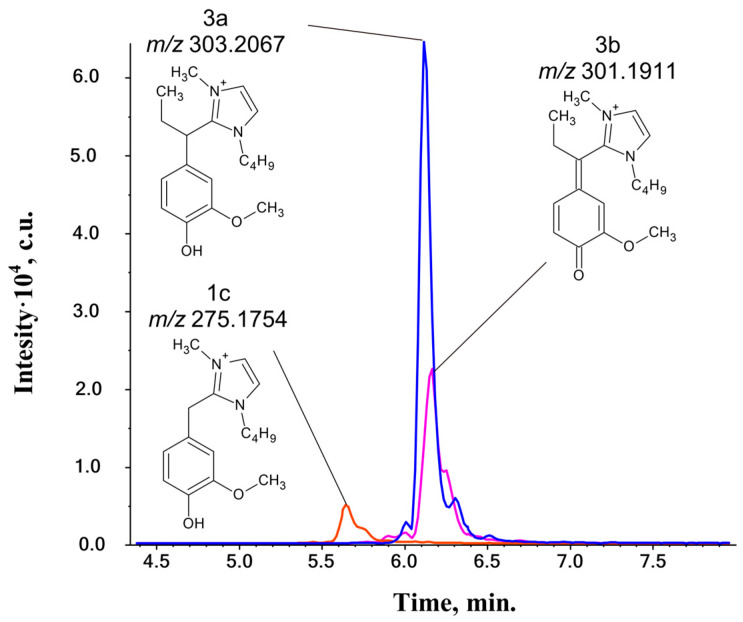
Exact mass-based extracted ion chromatograms (XICs) of the reaction products (**1c**, **3a**, **3b**) of isoeugenol with BmimOAc (150 °C, 300 min).

The possibility of interactions between the Bmim cation and aliphatic double C=C bonds has not yet been mentioned in the literature. To obtain its experimental confirmation or refutation, two lignin-related phenylpropane compounds, isoeugenol and eugenol, bearing conjugated and non-conjugated double bond in the propane chain, respectively, were chosen as the model substrates. HPLC-HRMS analysis of their reaction mixtures with BmimOAc (Figure 7) revealed the formation of two types of products with close retention times, designated as **3a** and **3b** (Table 3).

Both detected compounds are close in their structure and differ only by one DBE unit, which is the equivalent of two hydrogen atoms. Their tandem mass spectra (Supplementary Figure S17) appear to be similar, showing losses of methyl and ethyl radicals, butene molecules, or a combination of these. The observation of a [M-C_2_H_5_]^+^ fragment is only possible if the Bmim cation is bonded to the α-carbon atom of isoeugenol.

**Figure 8:**
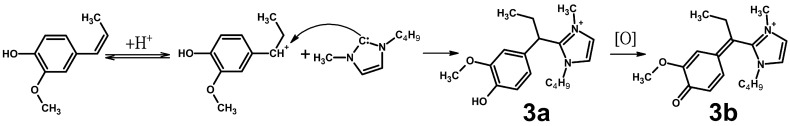
Formation of **3a**- and **3b**-type compounds in the reaction of Bmim with isoeugenol.

The corresponding cross-peaks in the HSQC and HMBC spectra were not detected due to the low concentrations of these compounds compared to the IL’s thermal degradation products (Supplementary Figures S18–S20). As in the case of the studied ketones, a shielding effect, caused by the presence of bounded bmim cation and imidazole derivatives, interferes with NMR identification of the structures. The **3a**-type product is assumed to be primary, while the **3b** type is a product of further transformation through the oxidation process (Figure 8).

The primary characteristic of the C=C bond is its susceptibility to electrophilic addition reactions. Therefore, the most reasonable explanation of the observed reaction pathway involves protonation in the first stage. The most stable carbocation would be the one with the positive charge at the benzyl position, although the formation of alternative species with the charged β-carbon atom cannot be fully excluded. The resulting carbocation is rapidly attacked by the NHC, resulting in the formation of the **3a** product type. It is worth noting that, in the case of eugenol, the carbocation formed in the first stage is readily rearranged through hydride-shift into a more stable benzyl carbocation, leading to the same products as those detected for isoeugenol. Interestingly, products formed during the treatment of vanillin with IL (**1a**–**1c**) were also found in the reaction mixture, indicating a gradual oxidation of the C=C double bond.

The temperature and duration of the IL treatment significantly affect the ratio of the formed compounds (Supplementary Table S4). At 80 °C, the **1a**–**1c** types predominate in the reaction mixture. The reaction with the double bond is observed starting at 120 °C, which results from the increase in the proportion of **3a** and **3b** products. The presence of a conjugated double bond in the isoeugenol structure promotes the earlier formation of the detected products. The **3a**-type compound is present in larger quantities than the **3b**-type. Upon prolonged heating, the relative amount of **3a** products gradually decreases while the **3b** levels increase. The maximum level of the **3a** type is observed after 120 min of heating at 150 °C. This phenomenon may be attributed to the gradual formation of various nitrogen-containing bases that prevent the formation of carbocation.

It should be noted that, contrary to expectations, the reactivity of lignin structural units with a double bond in transformations under the action of IL is rather high. Comparing the changes in the concentrations of the studied model compounds over time during the IL treatments (Supplementary Table S5), it can be concluded that, in terms of the rate and degree of conversion achieved, eugenol and isoeugenol are inferior only to aldehydes. Based on these data, it is suggested to arrange the functional groups of lignin in descending order of reactivity toward Bmim as follows: CHO >> C=C > OH_aliph_ > C=O.

## 3. Materials and Methods

### 3.1. Reagents and Materials

The chemicals used in this study were 1-butyl-3-methylimidazolium acetate (BASF quality, >95%), 4-Hydroxy-3-methoxybenzaldehyde (vanillin, 99%), 4-Hydroxy-3,5-dimethoxybenzaldehyde (syringaldehyde, 97%), 3,4-Dimethoxybenzaldehyde (veratraldehyde, 99%), 4-(Hydroxymethyl)-2-methoxyphenol (vanillin alcohol, 98%), 2-Methoxy-4-(prop-2-en-1-yl)phenol (eugenol, 99%), 2-Methoxy-4-(prop-1-en-1-yl)phenol (isoeugenol, 98%), 1-(4-Hydroxy-3-methoxyphenyl)ethan-1-one (acetovanillone, 99%), and 1-(4-Hydroxy-3,5-dimethoxyphenyl)ethan-1-one (acetosyringone, 99%) purchased from Sigma Aldrich (Steinheim, Germany). Deionized Type I water from the Milli-Q system (Millipore, Molsheim, France) and HPLC gradient grade acetonitrile (Cryochrom, St. Petersburg, Russia) were used for sample preparation and as the mobile phase components in HPLC analyses.

### 3.2. Reaction Mixtures and Sampling

A 10 mg sample of the model compound was placed in a 2 mL glass vial with air-tight screwcap and containing 1 g of preheated ionic liquid. Thermal treatment was performed under constant stirring at 80, 120, and 150 °C using a Reacti-Therm heating module (Thermo Fisher Scientific, Waltham, MA, USA). Simultaneously, thermal treatment of the IL blank sample was carried out in the same way. Aliquots (20 µL) were taken during the first 30 min at 10 min intervals, then at 30 min intervals for the next 2 h, and finally at 1 h intervals for the next 5 h. Immediately after sampling, each aliquot was diluted to 10 mL with an acetonitrile/water (20/80) mixture in a volumetric flask and subjected to HPLC-HRMS analysis. The initial sample (0 min) was a solution of the model compound (10 mg/mL) diluted to 10 mL and analyzed in the same manner.

To confirm the structure of the detected reaction products by NMR spectroscopy, 1 g of the IL was added to 100 mg of the model compound. The resulting mixture was heated in a glass vial for 24 h at the specified temperature. Subsequently, 500 μL of DMSO-d6 (99.8%, Deutero GmbH, Kastellaun, Germany) was added to the solution.

### 3.3. Analytical Methods

HPLC-HRMS analysis was carried out using a TripleTOF 5600+ quadrupole time-of-flight (Q-TOF) high-resolution mass spectrometer (AB Sciex, Concord, ON, Canada) and an LC-30 Nexera HPLC system (Shimadzu, Kyoto, Japan). Analytes were ionized in the positive electrospray ionization (ESI+) mode with the following ion source parameters: curtain gas (CUR) pressure 25 psi; nebulizing (GS1) and drying (GS2) gas pressure 40 psi, capillary voltage 5500 V, source temperature 300 °C, and declustering potential 100 V. Scanning range of *m*/*z* 100–1000 and *m*/*z* 20–1000 was set in MS and MS/MS experiments, respectively. Tandem mass spectra were acquired in an information-dependent (IDA) mode for the precursor ions with signal intensities exceeding 100 cps. Collision-induced dissociation (CID) was used with a collision energy (CE) of 40 eV and a spread of 20 eV. Chromatographic separation was carried out in a gradient elution mode on a Nucleodur PFP column, 150 × 3 mm, 1.8 μm particle size (Macherey-Nagel, Duren, Germany), with pentafluorophenyl stationary phase. The mobile phase consisted of water (A) and acetonitrile (B), both containing 0.1% of formic acid. The following gradient program was used: 0–1 min—20% B, 1–10 min—linear increase to 100% B, 10–15 min—100% B. Injection volume was 2 µL, mobile phase flow rate was 0.4 mL/min, and column thermostat temperature—40 °C.

NMR spectra were recorded on an AVANCE III NMR spectrometer (Bruker, Ettlingen, Germany) with a working frequency for protons of 600 MHz. The ^1^H-^13^C HSQC spectra were recorded using the following parameters: temperature—298 K, spectral window width ~13 ppm for F2 and ~200 ppm for F1 with a number of accumulations—1024 × 256, number of scans—8, delay time between pulses (D1)—2.0 s. The following parameters were used to record ^1^H-^13^C HMBC spectra: temperature—298 K, spectral window width ~12 ppm for F2 and ~ 240 ppm for F1 with accumulation number—2048 × 512, number of scans—8, time delay between pulses (D1)—1.2 s.

## 4. Conclusions

The obtained experimental data clearly indicate that, at temperatures commonly used in IL-based biorefining technologies, the main functional groups of lignin macromolecules (aldehyde, ketone, conjugated and non-conjugated carbon–carbon double bonds, aliphatic hydroxyl) may react with the Bmim cation of an IL, resulting in the formation of various nitrogen-containing products. The latter possess, in their structures, covalently bonded Bmim residues and are formed through the nucleophilic addition of the N-heterocyclic carbene (deprotonated Bmim cation) to electron-deficient groups, followed by the transformation of the resulting intermediates. The reactivity of lignin functional groups in interactions with NHC decreases in the order CHO >> C=C > OH > C=O and is greatly affected by the temperature and dissolved oxygen. At higher temperatures (120–150 °C), IL’s thermal degradation products, primarily aliphatic amines, accumulate in the solution and act as additional reactive species toward lignin, further complicating the range of products formed.

These findings provide an explanation for the substantial nitrogen content in IL lignin preparations isolated from plant materials using N,N′-dialkylimidazolium, which was previously described in the literature. The obtained results made it possible to answer the question posed in this article’s title and to assert that 1-butyl-3-methylimidazolium-based ILs act as active reagents with respect to lignin during the dissolution of lignocellulose. Covalent nitrogen bonding increases the solubility of the biopolymer and prevents undesirable condensation processes, which may largely determine the efficiency of ILs in biorefining processes. On the other hand, the formation of a large number of nitrogen-containing lignin transformation products with unknown toxicities casts in doubt the classifying of some ILs as green solvents in biorefining processes and the possibility of their efficient regeneration.

The formation of NHC, and thus an IL’s reactivity, may strongly depend on the nature of the anion and the presence of various modifiers or additives in the solution. Therefore, further research should be aimed at studying the effects of these factors on the transformation of lignin substances and developing, on this basis, approaches to reducing the negative effects of using ILs for fractionating lignocellulose under the biorefinery concept.

## Figures and Tables

**Table 1:** Products of reaction of aldehydes and vanillin alcohol with BmimOAc detected by HPLC-HRMS.

Substrate	Product Type	Formula	Retention Time, min	*m*/*z*	Δ*m*/*z*, ppm
Vanillin/ Vanillin Alcohol	**1a**	[C_16_H_23_N_2_O_3_]^+^	5.3	291.1700	1.0
	**1b**	[C_16_H_21_N_2_O_3_]^+^	5.8	289.1552	1.7
	**1c**	[C_16_H_23_N_2_O_2_]^+^	5.5	275.1749	1.8
	**1d**	[C_17_H_24_N_3_O_2_]^+^	5.6	302.1867	1.3
	**1e**	[C_20_H_30_N_3_O_2_]^+^	6.5	344.2322	3.2
Syringaldehyde	**1a**	[C_17_H_25_N_2_O_4_]^+^	5.3	321.1807	0.6
	**1b**	[C_17_H_23_N_2_O_4_]^+^	5.8	319.1656	1.3
	**1c**	[C_17_H_25_N_2_O_3_]^+^	5.5	305.1855	1.6
	**1d**	[C_18_H_26_N_3_O_3_]^+^	5.7	332.1962	2.1
	**1e**	[C_21_H_32_N_3_O_3_]^+^	6.5	374.2452	3.7
Veratraldehyde	**1a**	[C_17_H_25_N_2_O_3_]^+^	5.5	305.1862	0.7
	**1c**	[C_17_H_25_N_2_O_2_]^+^	6.0	289.1906	1.7

**Table 2:** Products of reaction of aromatic ketones with BmimOAc detected by HPLC-HRMS.

Substrate	Product Type	Formula	Retention Time, min	*m*/*z*	Δ*m*/*z*, ppm
Acetovanillone	**2a**	[C_17_H_25_N_2_O_3_]^+^	5.2	305.1854	2.0
	**2b**	[C_17_H_23_N_2_O_3_]^+^	6.8	303.1701	0.7
	**2c**	[C_18_H_25_N_2_O_3_]^+^	6.0	317.1854	1.9
	**2d**	[C_21_H_31_N_2_O_3_]^+^	6.1	359.2327	0.6
Acetosyringone	**2a**	[C_18_H_27_N_2_O_4_]^+^	5.4	335.1962	0.9
	**2b**	[C_18_H_25_N_2_O_4_]^+^	6.3	333.1807	0.6
	**2c**	[C_19_H_27_N_2_O_4_]^+^	6.0	347.1950	4.3
	**2d**	[C_22_H_33_N_2_O_4_]^+^	6.6	389.2427	1.8

**Table 3:** Products of reaction of phenolic compounds bearing aliphatic double C=C bond with BmimOAc detected by HPLC-HRMS.

Substrate	Product Type	Formula	Retention Time, min	*m*/*z*	Δ*m*/*z*, ppm
Isoeugenol	**3a**	[C_18_H_27_N_2_O_2_]^+^	6.12	303.2071	1.3
	**3b**	[C_18_H_25_N_2_O_2_]^+^	6.13	301.1896	5.0
Eugenol	**3a**	[C_18_H_27_N_2_O_2_]^+^	6.12	303.2058	3.0
	**3b**	[C_18_H_25_N_2_O_2_]^+^	6.13	301.1907	1.3

Substrate

Product Type

Formula

Retention Time, min

*m*/*z*

Δ*m*/*z*, ppm

## Data Availability

The data presented in this study are available in the article and Supplementary Materials.

## Supplementary Materials

The following supporting information can be downloaded at: https://www.mdpi.com/article/10.3390/ijms252312623/s1.
