# Supplementary material for: 1-Butyl-3-methylimidazolium-Based Ionic Liquid in Biomass Fractionation—Green Solvent or Active Reagent Toward Lignin Compounds?
Source: Int J Mol Sci. 2024 Nov 25;25(23):12623. doi: 10.3390/ijms252312623 (PMC11641608; doi:10.3390/ijms252312623)
Supplement: Supplementary file 1 [file ijms-25-12623-s001.zip › ijms-3316020-supplementary.pdf]

Supplementary Materials

1-Butyl-3-methylimidazolium-Based Ionic Liquid in Biomass Fractionation—Green Solvent or Active Reagent Toward Lignin Compounds?

Artyom V. Belesov [1], Dmitrii M. Mazur [2,3], Anna V. Faleva [1], Ilya S. Varsegov [1], Ilya I. Pikovskoi [1], Nikolay V. Ulyanovskii [1] and Dmitry S. Kosyakov [1,*]

[1] Core Facility Center 'Arktika', Northern (Arctic) Federal University, Arkhangelsk 163002, Russia; a.belesov@narfu.ru (A.V.B.); a.bezumova@narfu.ru (A.V.F.); i.varsegov@narfu.ru (I.S.V.); i.pikovskoj@narfu.ru (I.I.P.); n.ulyanovsky@narfu.ru (N.V.U.)

[2] Department of Materials Science, MSU-BIT University, Shenzhen 517182, China; neodmitrii@gmail.com

[3] Department of Organic Chemistry, Lomonosov Moscow State University, Moscow 119991, Russia

* Correspondence: d.kosyakov@narfu.ru

Contents:

Table S1. Relative contents (% of the total chromatographic peak area) of the reaction products of aromatic aldehydes and vanillin alcohol with BmimOAc at various temperatures and treatment durations.
Table S2. Effect of oxygen on the formation of reaction products of vanillin alcohol with BmimOAc.
Table S3. Relative contents (% of the total chromatographic peak area) of reaction products of aromatic ketones with BmimOAc at various temperatures and treatment durations.
Table S4. Relative contents (% of the total chromatographic peak area) of reaction products of phenylpropane compounds, bearing C=C double bond, with BmimOAc at various temperatures and treatment durations.
Table S5. Concentrations (mg/mL) of lignin model compounds in reaction mixtures during treatments with BmimOAc
Figure S1. Tandem (CID) mass-spectra of aromatic aldehyde 1a-type reaction products.
Figure S2. Tandem (CID) mass-spectra of aromatic aldehyde 1b-type reaction products.
Figure S3. Tandem (CID) mass-spectra of aromatic aldehyde 1c-type reaction products.
Figure S4. Tandem (CID) mass-spectra of aromatic aldehyde 1e-type reaction products.
Figure S5. 2D HSQC/HBMC NMR spectra and structure elucidation of the reaction product 1a, where colors denote: red – HMBC spectra; blue (CH_3-CH signals) and violet (CH_2 signals) – HSQC spectra.
Figure S6. 2D HSQC/HBMC NMR spectra and structure elucidation of the reaction product 1c, where colors denote: red – HMBC spectra; blue (CH_3-CH signals) and violet (CH_2 signals) – HSQC spectra.
Figure S7. 2D HSQC NMR spectrum of the vanillin-BmimOAc reaction mixture
Figure S8. 2D HMBC NMR spectrum of the vanillin-BmimOAc reaction mixture
Figure S9. Tandem (CID) mass-spectra of aromatic ketone 2a-type reaction products.
Figure S10. Tandem (CID) mass-spectra of aromatic ketone 2b-type reaction products
Figure S11. Tandem (CID) mass-spectra of aromatic ketone 2c-type reaction products.
Figure S12. Tandem (CID) mass-spectra of aromatic ketone 2d-type reaction products.
Figure S13. 2D HSQC NMR spectrum of the acetovanillone-BmimOAc reaction mixture.
Figure S14. 2D HSQC NMR calculated spectrum of the acetovanillone reaction product 2b.
Figure S15. 2D HSQC NMR calculated spectrum of the acetovanillone reaction product 2c.
Figure S16. 2D HSQC NMR calculated spectrum of the acetovanillone reaction product 2d.
Figure S17. Tandem (CID) mass-spectra of isoeugenol 3a,b reaction products.
Figure S18. 2D HSQC NMR spectrum of the isoeugenol-BmimOAc reaction mixture.
Figure S19. 2D HSQC NMR calculated spectrum and structure of the reaction product 3a.

Figure S20. 2D HSQC NMR calculated spectrum and structure of the reaction product 3b.

Table S1. Relative contents (% of the total chromatographic peak area) of reaction products of aromatic aldehydes and vanillin alcohol with BmimOAc at various temperatures and treatment durations.

**Substrate**	**Product type**	**Temperature, °C**	**Treatment duration, min**								
			**10**	**20**	**30**	**60**	**90**	**120**	**180**	**240**	**300**
Vanillin	1a	80	58	60	53	42	41	39	31	21	19
		120	88	83	76	61	56	50	40	35	35
		150	67	59	52	37	14	12	6	2	1
	1b	80	42	40	46	56	57	58	66	74	76
		120	9	13	17	20	21	20	20	20	17
		150	4	6	6	6	4	2	1	0	0
	1c	80	0	0	0	0	0	0	0	0	0
		120	2	3	4	12	13	17	22	23	26
		150	22	24	29	43	67	80	91	96	97
	1d	80	0	0	0	1	1	1	1	2	2
		120	0	0	1	3	5	7	11	15	17
		150	4	8	9	10	9	4	1	1	1
	1e	80	0	0	1	1	1	2	2	3	2
		120	1	1	2	5	5	6	6	6	5
		150	2	3	3	5	6	2	1	0	0
Syringaldehyde	1a	80	57	71	73	61	52	45	38	29	28
		120	83	73	64	48	41	38	28	23	20
		150	45	35	28	14	10	8	4	4	4
	1b	80	4	12	17	27	35	41	45	53	50
		120	8	10	12	15	12	11	8	6	5
		150	1	2	2	1	1	0	0	0	0
	1c	80	39	17	8	8	8	6	5	3	2
		120	1	0	0	1	3	3	8	10	12
		150	42	46	49	67	71	72	85	85	89
	1d	80	0	0	0	1	2	2	3	6	5
		120	2	6	9	20	29	34	45	51	52
		150	8	12	16	14	14	15	8	8	5
	1e	80	0	1	1	3	3	6	9	10	15
		120	6	11	14	16	14	14	11	9	10
		150	5	5	5	4	4	4	2	3	2
Vanillin alcohol	1a	80	1	1	1	1	1	1	1	2	2
		120	0	0	0	1	1	1	3	7	19
		150	0	1	1	1	1	1	1	1	0
	1b	80	32	20	19	16	13	10	8	7	7
		120	2	4	6	8	9	11	13	27	44
		150	2	1	0	0	0	0	0	0	0
	1c	80	67	79	80	83	86	89	90	90	89
		120	97	96	94	91	89	85	78	55	19
		150	98	98	99	98	97	97	97	97	98
	1d	80	0	0	0	0	0	0	0	0	1
		120	0	0	0	0	0	1	4	7	10
		150	0	0	0	1	1	1	1	1	1
	1e	80	0	0	0	0	0	0	0	0	1
		120	0	0	0	0	1	1	2	3	9
		150	0	0	0	0	0	1	1	1	0
Veratraldehyde	1a	80	100	100	100	100	100	100	100	99	99
		120	100	99	98	93	87	82	74	67	45
		150	95	96	94	89	84	74	47	34	25
	1c	80	0	0	0	0	0	0	0	1	1

		120	0	1	1	6	12	16	24	30	53
		150	5	3	5	10	15	25	51	64	73

Table S2. Effect of oxygen on the formation of reaction products of vanillin alcohol with BmimOAc

**Product type**	**Chromatographic peak area, arb. units**	
	**Inert environment (argon)**	**Atmospheric air**
1a	60	501
1b	0	1020
1c	14500	6550

Table S3. Relative contents (% of the total chromatographic peak area) of reaction products of aromatic ketones with BmimOAc at various temperatures and treatment durations.

**Substrate**	**Product type**	**Temperature, °C**	**Treatment duration, min**								
			**10**	**20**	**30**	**60**	**90**	**120**	**180**	**240**	**300**
Acetovanillone	2a	80	100	100	100	83	77	76	75	77	74
		120	58	60	58	53	51	47	49	48	44
		150	78	71	52	26	17	9	6	4	2
	2b	80	0	0	0	17	23	24	25	23	26
		120	42	40	42	44	45	48	45	44	49
		150	12	14	18	17	13	14	15	20	26
	2c	80	0	0	0	0	0	0	0	0	0
		120	0	0	0	3	4	5	6	8	8
		150	9	10	19	47	50	56	57	57	52
	2d	80	0	0	0	0	0	0	0	0	0
		120	0	0	0	0	0	0	0	0	0
		150	2	5	10	10	20	21	21	19	20
Acetosyringone	2a	80	0	0	0	0	0	0	0	0	0
		120	50	57	55	41	36	38	36	36	33
		150	40	12	9	5	4	4	2	1	1
	2b	80	0	0	0	0	0	0	0	0	0
		120	50	43	45	59	64	62	64	64	67
		150	40	38	43	35	37	33	20	13	9
	2c	80	0	0	0	0	0	0	0	0	0
		120	0	0	0	0	0	0	0	0	0
		150	20	12	15	13	11	12	18	17	22
	2d	80	0	0	0	0	0	0	0	0	0
		120	0	0	0	0	0	0	0	0	0
		150	0	38	33	47	48	51	60	69	69

Table S4. Relative contents (% of the total chromatographic peak area) of reaction products of phenylpropane compounds, bearing C=C double bond, with BmimOAc at various temperatures and treatment durations.

Substrate	Product type	Temperature, °C	Treatment duration, min								
			**10**	**20**	**30**	**10**	**90**	**120**	**10**	**240**	**300**
		80	19	21	16	10	12	12	10	9	9
	1a	120	5	5	5	5	5	5	6	7	7
		150	1	1	1	1	1	1	1	1	1
		80	63	61	70	79	77	78	80	83	81
	1b	120	44	39	31	20	14	13	9	9	9
		150	3	2	1	1	0	0	0	0	0
		80	18	15	12	7	7	5	4	4	4
Isoeugenol	1c	120	21	17	16	16	14	15	16	17	19
		150	6	4	4	4	4	5	5	6	6
		80	1	3	3	4	4	4	6	5	7
	3a	120	31	39	48	59	66	65	68	64	63
		150	88	87	88	82	83	79	59	52	50
		80	0	0	0	0	0	0	0	0	0
	3b	120	0	0	0	0	1	1	2	2	2
		150	2	6	6	12	12	15	35	41	43
		80	36	32	28	23	17	16	11	12	13
	1a	120	20	18	15	17	14	13	14	13	12
		150	14	5	4	4	4	4	4	4	4
		80	27	32	39	40	57	59	72	71	73
	1b	120	26	33	29	22	25	22	16	12	12
		150	14	8	7	1	0	0	0	0	0
		80	37	36	34	36	26	25	17	18	14
Eugenol	1c	120	51	45	50	50	47	50	51	53	48
		150	35	18	18	14	13	14	16	26	28
		80	0	0	0	0	0	0	0	0	0
	3a	120	0	2	3	5	6	8	11	13	15
		150	32	65	64	73	76	76	67	53	49
		80	0	0	0	0	0	0	0	0	0
	3b	120	3	3	4	6	7	7	7	9	13
		150	5	4	6	8	7	7	13	17	19

Table S5. Concentrations (mg/mL) of lignin model compounds in reaction mixtures during treatments with BmimOAc

Substrate	Temperature, °C	Treatment duration, min									
		0	10	20	30	60	90	120	180	240	300
Vanillin	80	10.0	8.3	7.8	7.2	6.9	6.6	6.1	5.9	5.5	5.1
	120	10.0	7.2	6.9	6.8	6.3	5.8	5.4	5.0	5.0	4.6
	150	10.0	6.3	5.9	5.1	4.4	4.1	3.6	3.3	2.8	2.4
Syringaldehyde	80	10.0	9.5	9.2	8.5	8.0	7.9	7.8	7.6	7.2	7.0
	120	10.0	9.3	9.0	8.3	8.4	8.2	7.9	7.2	6.8	6.3
	150	10.0	9.4	9.1	8.3	7.3	6.9	5.5	4.8	2.7	2.5
Veratraldehyde	80	10.0	0.5	0.5	0.5	0.4	0.4	0.4	0.4	0.3	0.3
	120	10.0	1.0	1.0	0.9	0.8	0.8	0.8	0.7	0.4	0.2
	150	10.0	2.3	2.1	2.0	1.5	0.9	0.5	0.3	0.1	0.1
Vanillin alcohol	80	10.0	8.9	8.6	8.0	7.7	7.6	7.2	6.9	6.5	6.2
	120	10.0	8.0	7.8	7.5	7.2	6.8	6.2	6.0	5.7	5.4
	150	10.0	7.6	6.5	6.2	5.9	5.5	5.1	4.8	4.5	4.2
Acetovanillone	80	10.0	9.9	9.8	9.8	9.8	9.5	9.1	8.9	8.7	8.6
	120	10.0	9.9	9.5	9.4	9.2	8.7	8.6	8.3	8.3	8.2
	150	10.0	8.8	8.3	7.2	7.2	6.5	4.6	3.1	2.3	2.1
Acetosyringone	80	10.0	9.9	9.7	9.5	9.3	8.9	8.6	8.4	8.1	7.9
	120	10.0	9.7	9.5	9.2	9.2	8.8	8.5	8.3	7.9	7.7
	150	10.0	9.6	9.4	9.0	8.7	8.5	8.2	7.9	7.7	7.5
Eugenol	80	10.0	8.9	8.8	8.7	8.3	7.9	7.0	6.8	6.5	6.1
	120	10.0	8.6	8.5	8.3	8.0	7.1	6.8	6.2	5.6	4.4
	150	10.0	8.4	8.1	8.0	7.3	6.7	5.7	5.4	4.6	2.6
Isoeugenol	80	10.0	8.6	7.6	7.6	6.8	6.5	5.9	5.2	5.0	4.6
	120	10.0	7.9	7.3	7.1	6.6	6.5	5.8	5.0	4.2	4.0
	150	10.0	7.8	7.2	7.1	6.5	6.2	5.2	4.5	3.6	2.6

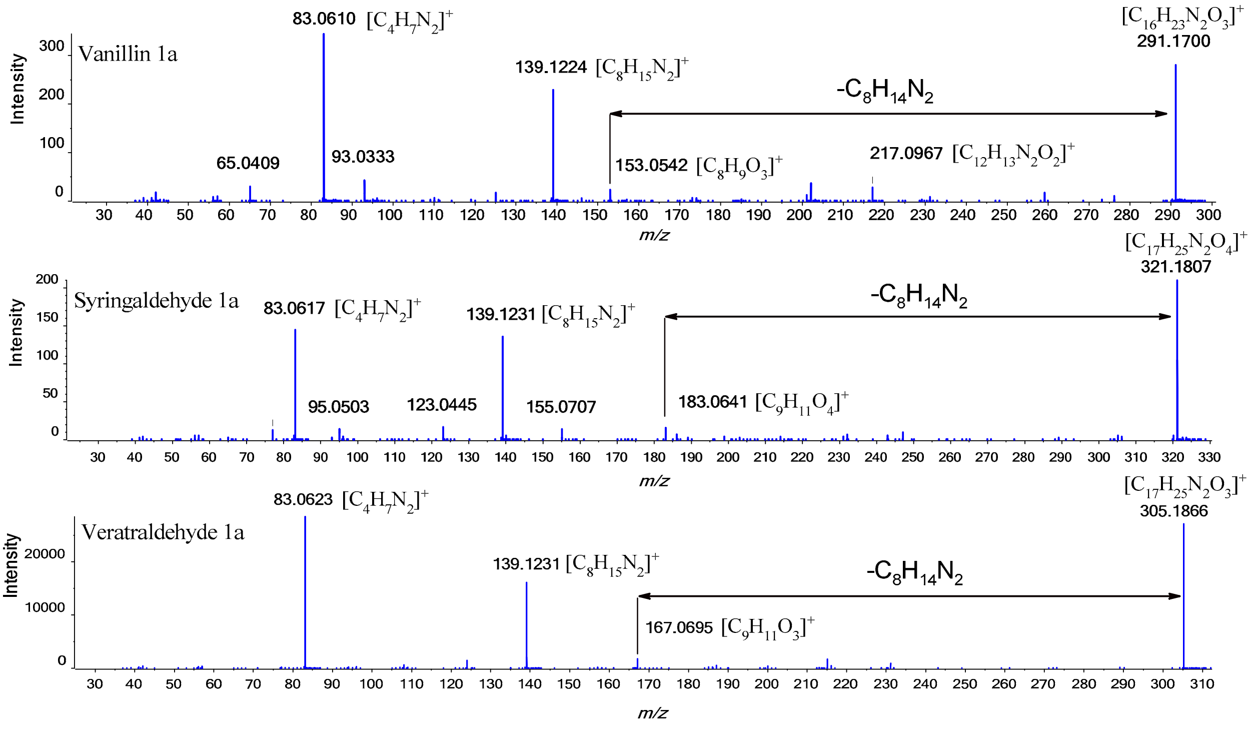

Figure S1. Tandem (CID) mass-spectra of aromatic aldehyde 1a-type reaction products.

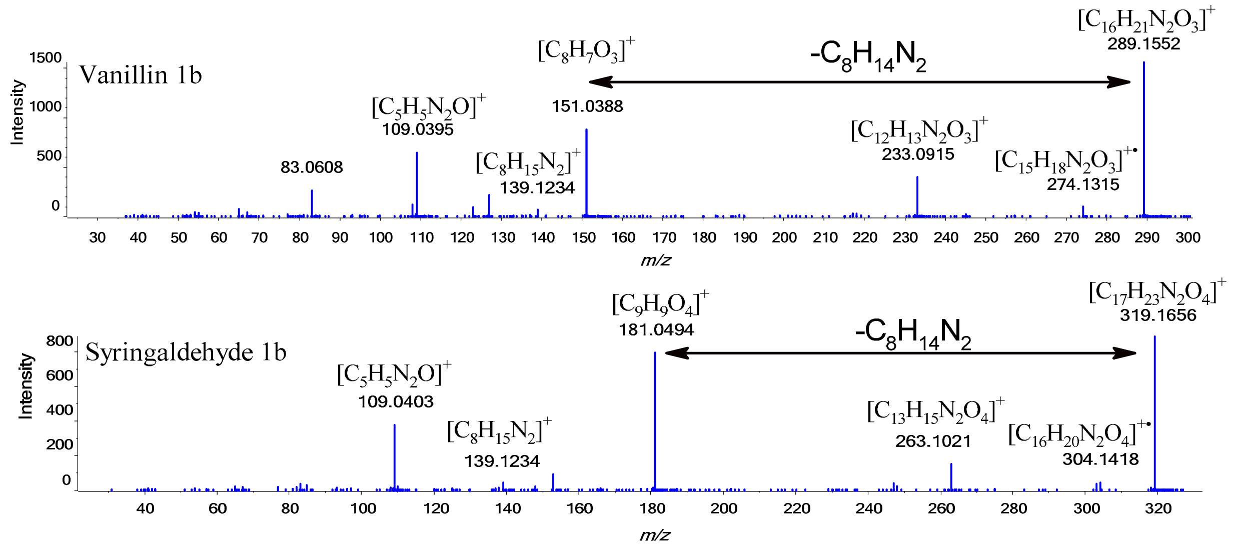

Figure S2. Tandem (CID) mass-spectra of aromatic aldehyde 1b-type reaction products.

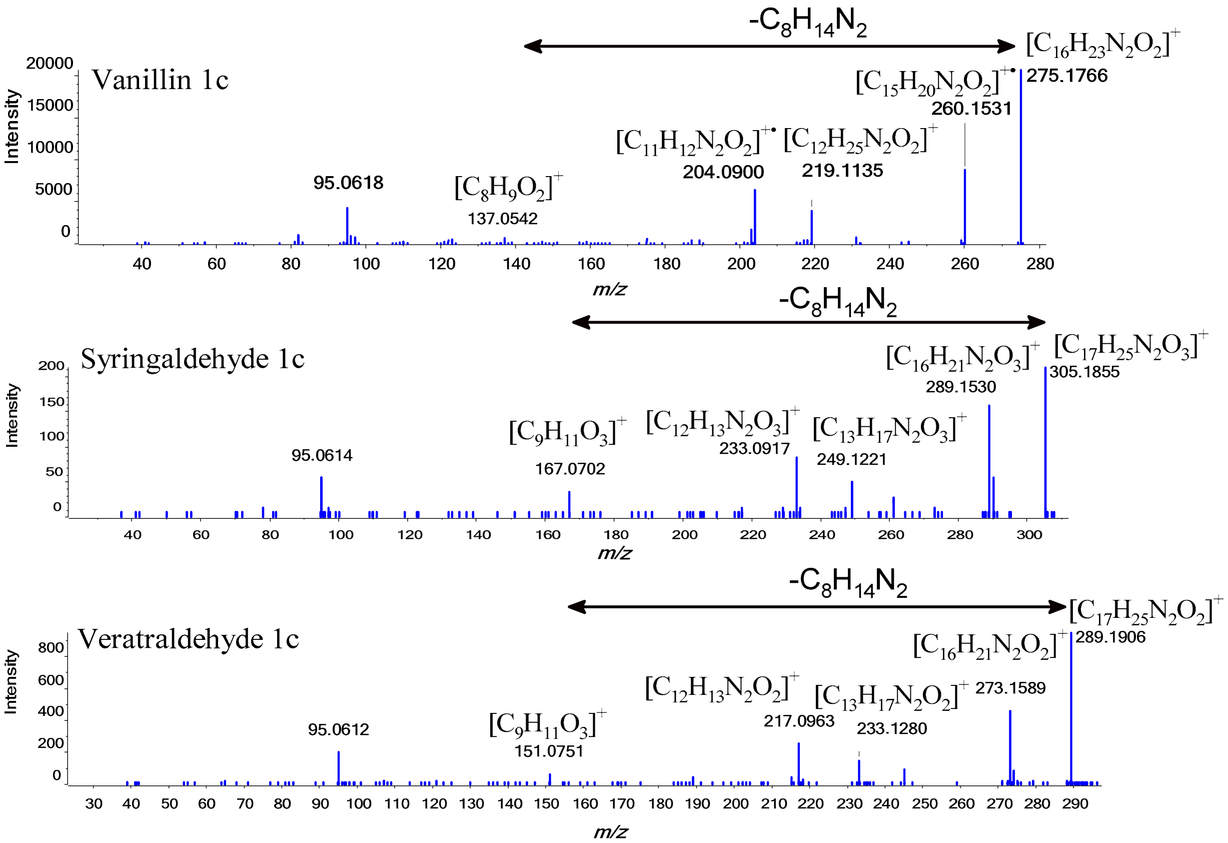

Figure S3. Tandem (CID) mass-spectra of aromatic aldehyde 1c-type reaction products.

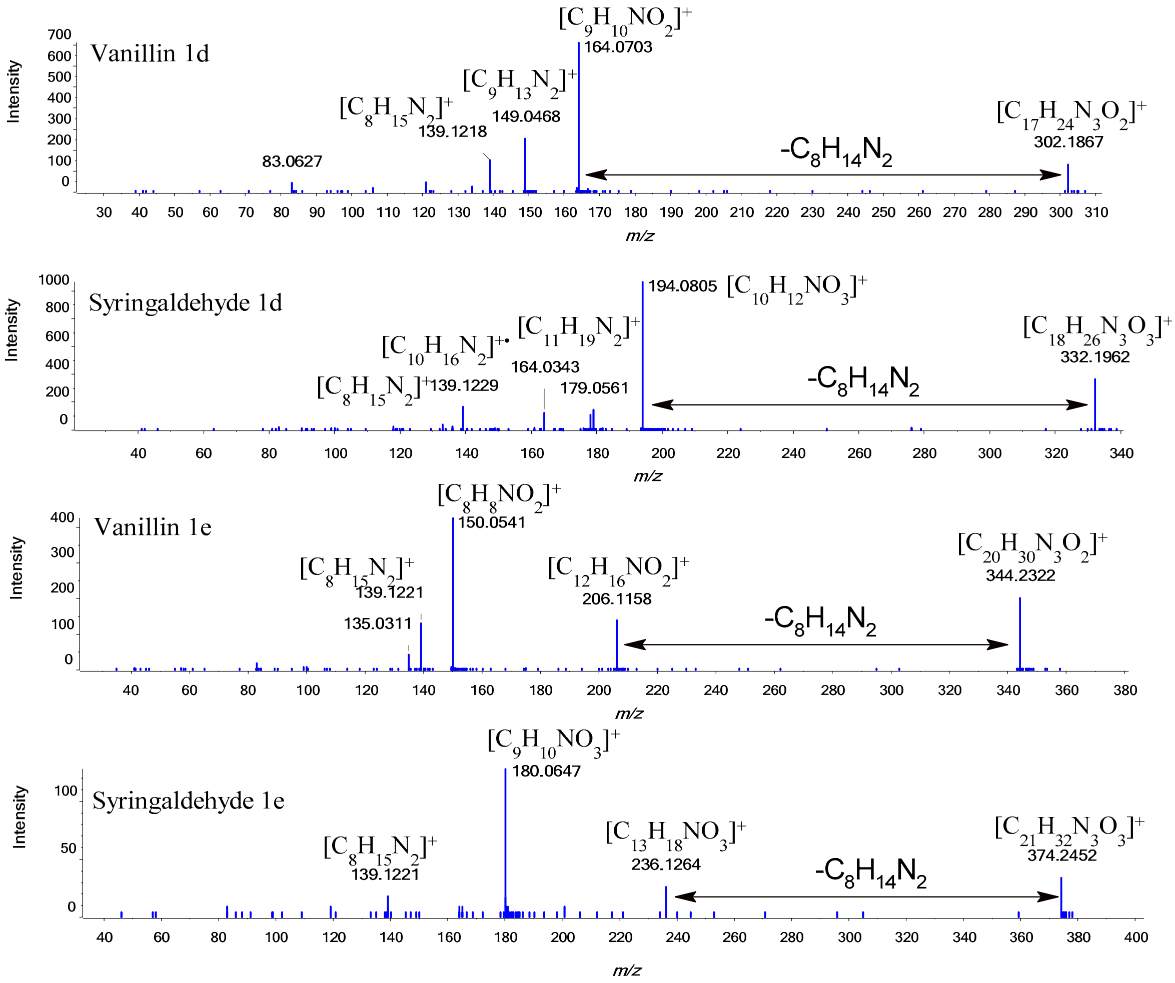

Figure S4. Tandem (CID) mass-spectra of aromatic aldehyde 1e-type reaction products.

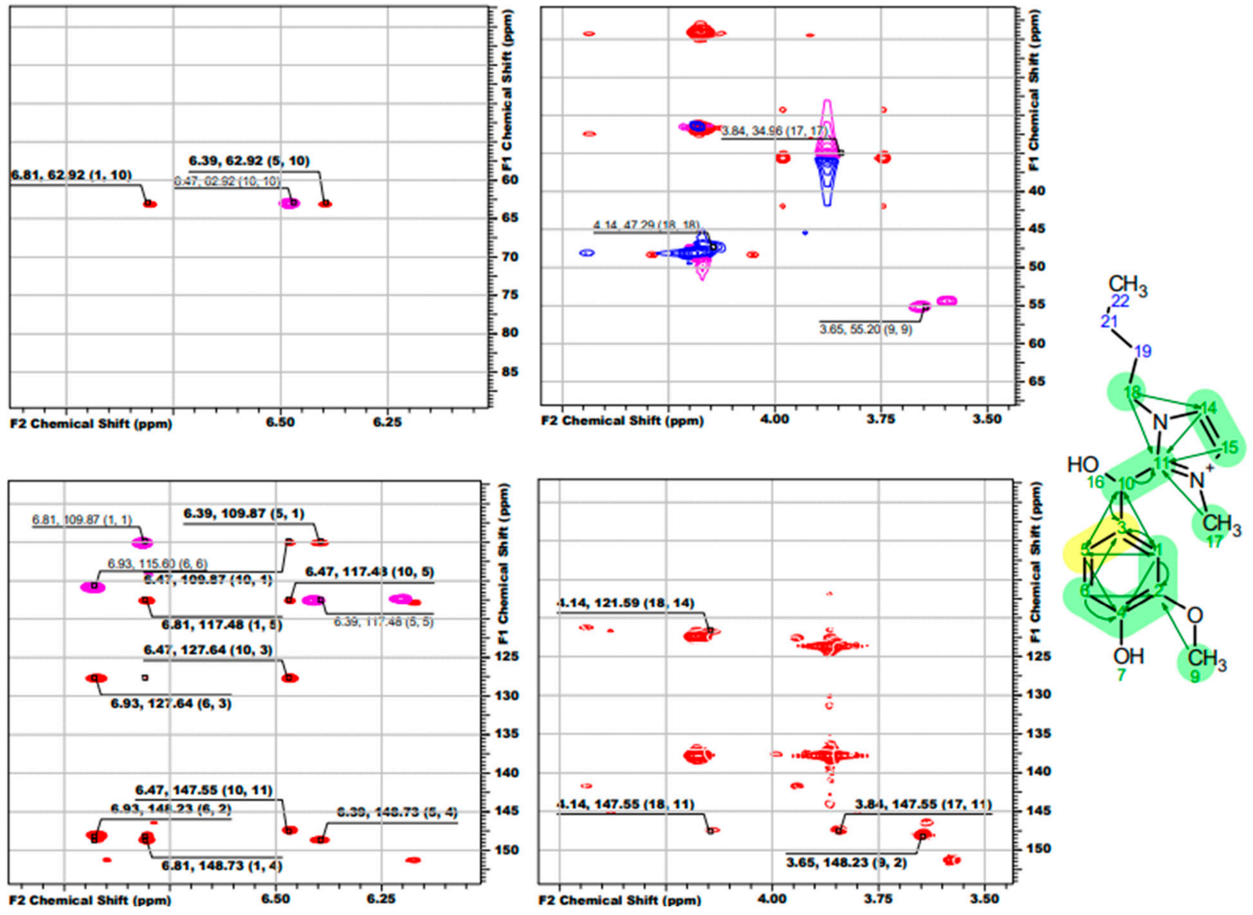

Figure S5. 2D HSQC/HBMC NMR spectra and structure elucidation of the reaction product 1a, where colors denote: red – HMBC spectra; blue (CH_3-CH signals) and violet (CH_2 signals) – HSQC spectra.

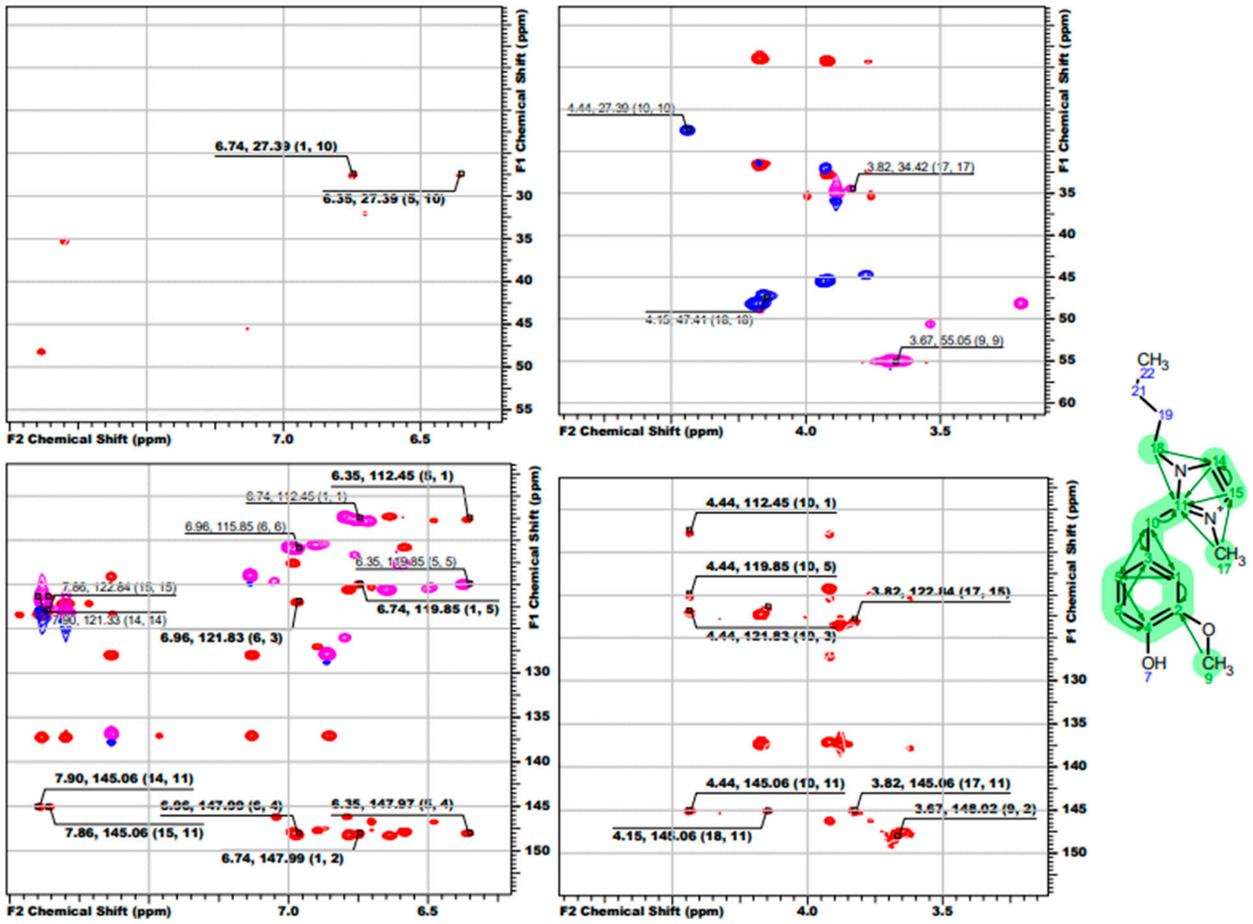

Figure S6. 2D HSQC/HBMC NMR spectra and structure elucidation of the reaction product 1c, where colors denote: red – HMBC spectra; blue (CH_3-CH signals) and violet (CH_2 signals) – HSQC spectra.

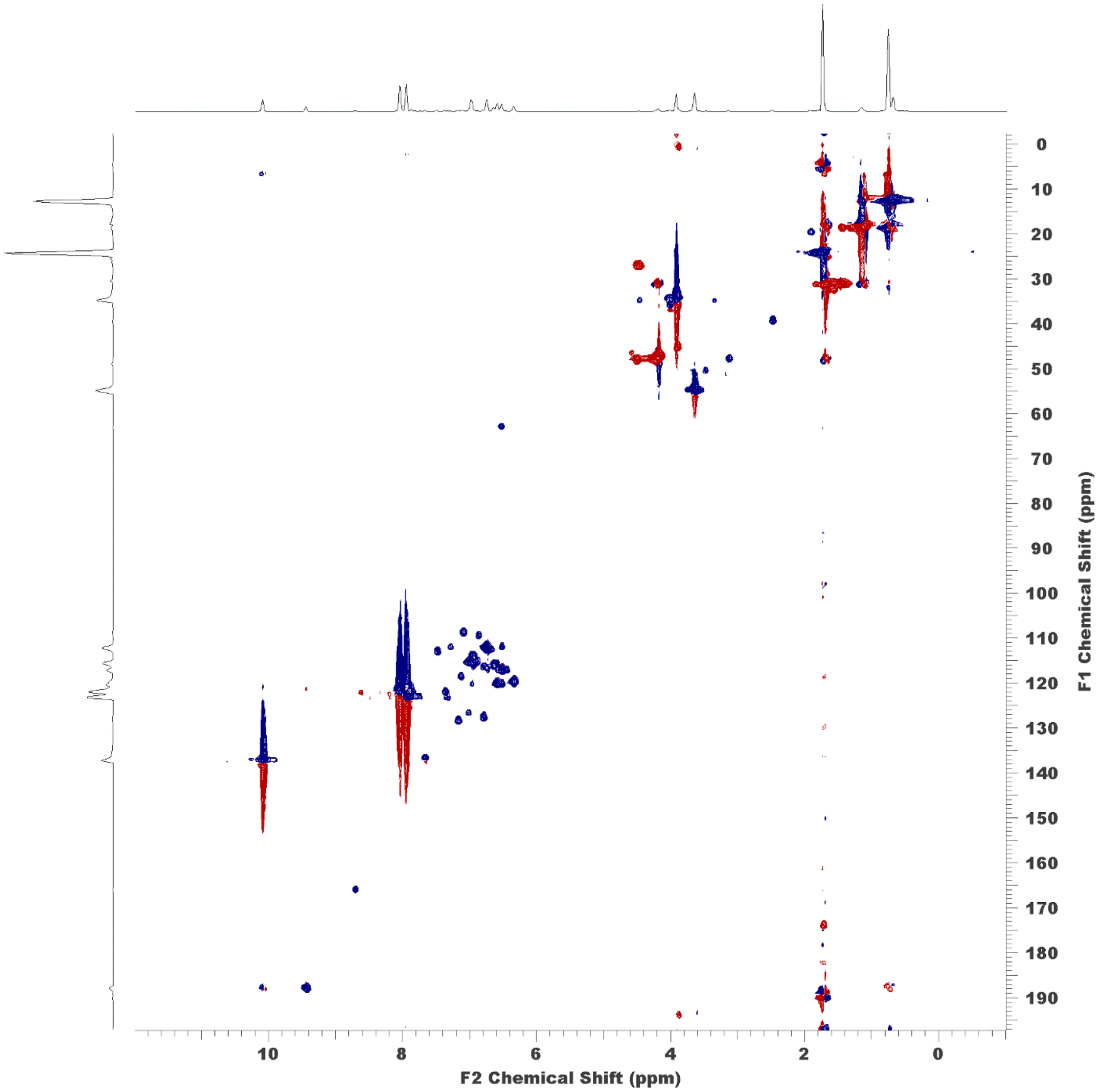

Figure S7. 2D HSQC NMR spectrum of the vanillin-BmimOAc reaction mixture

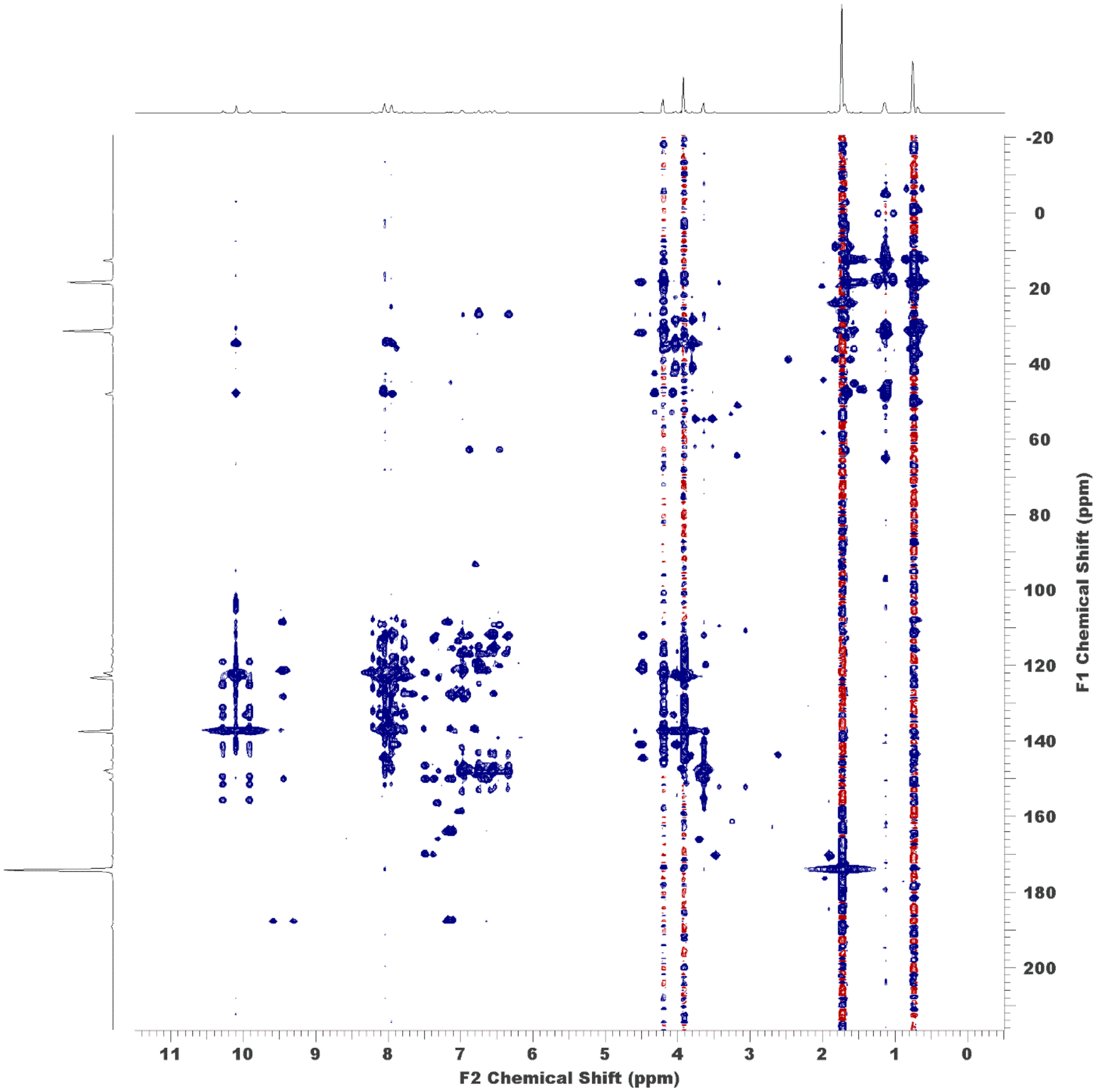

Figure S8. 2D HMBC NMR spectrum of the vanillin-BmimOAc reaction mixture

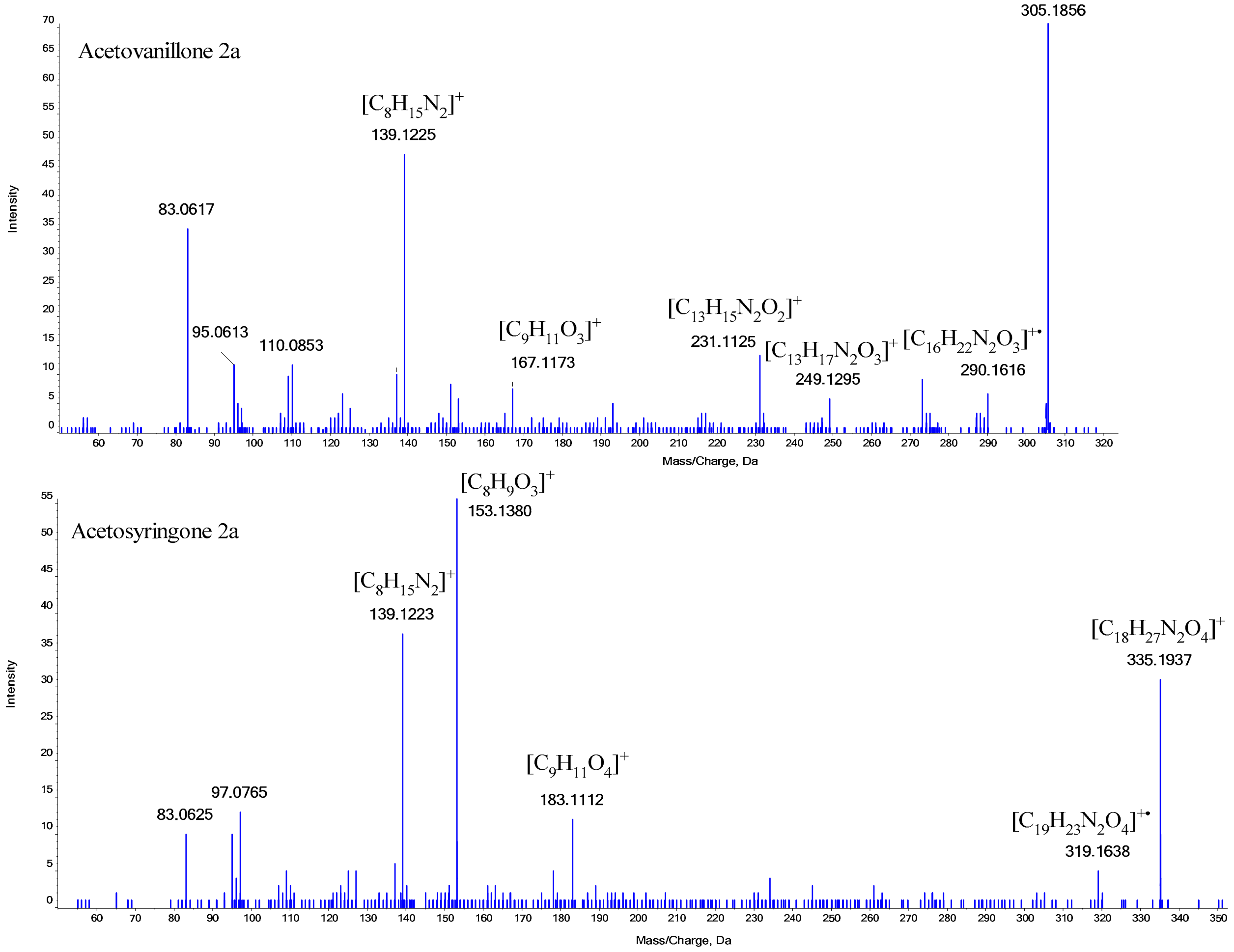

Figure S9. Tandem (CID) mass-spectra of aromatic ketone 2a-type reaction products.

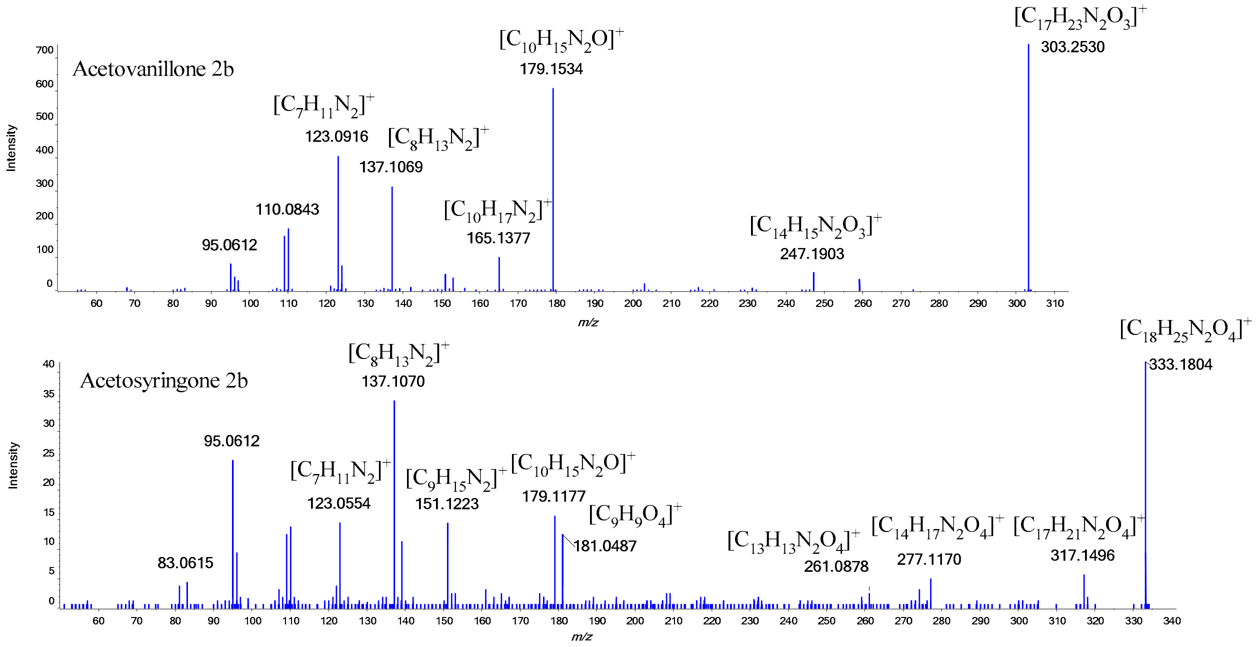

Figure S10. Tandem (CID) mass-spectra of aromatic ketone 2b-type reaction products.

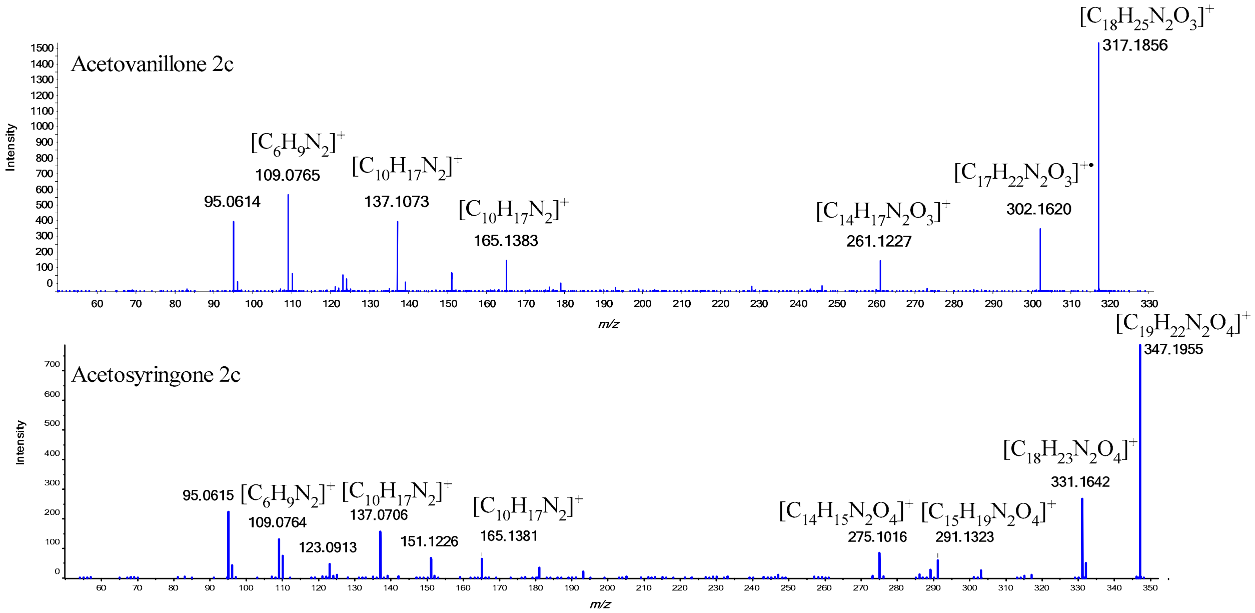

Figure S11. Tandem (CID) mass-spectra of aromatic ketone 2c-type reaction products.

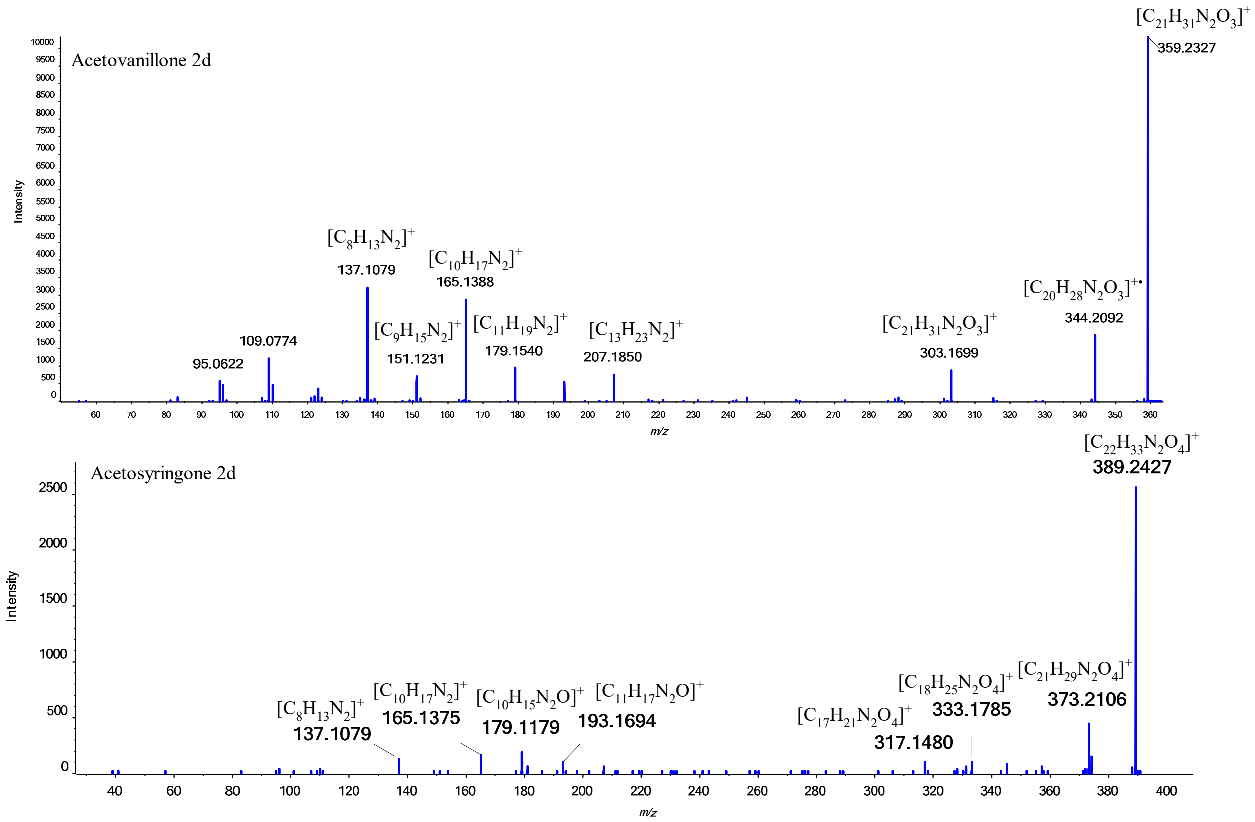

Figure S12. Tandem (CID) mass-spectra of aromatic ketone 2d-type reaction products.

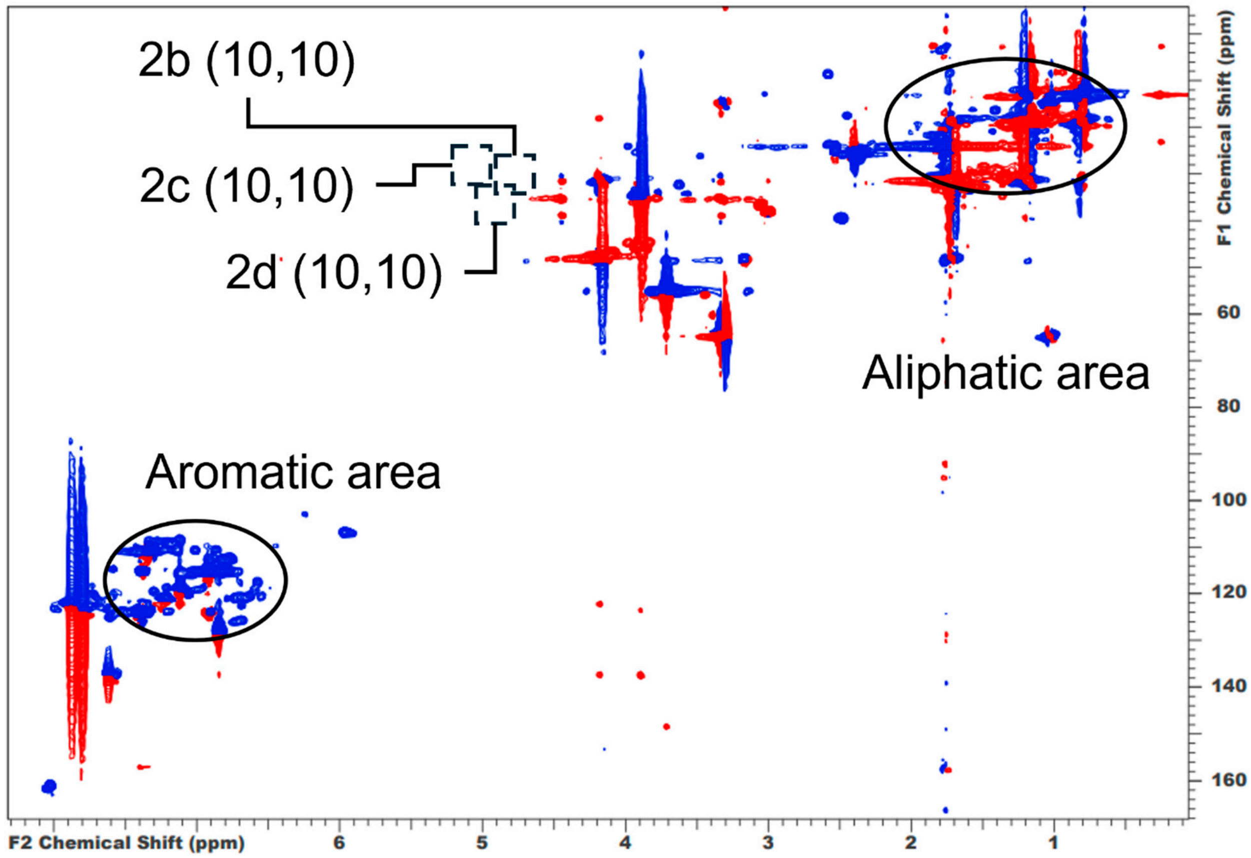

Figure S13. 2D HSQC NMR spectrum of the acetovanillone-BmimOAc reaction mixture.

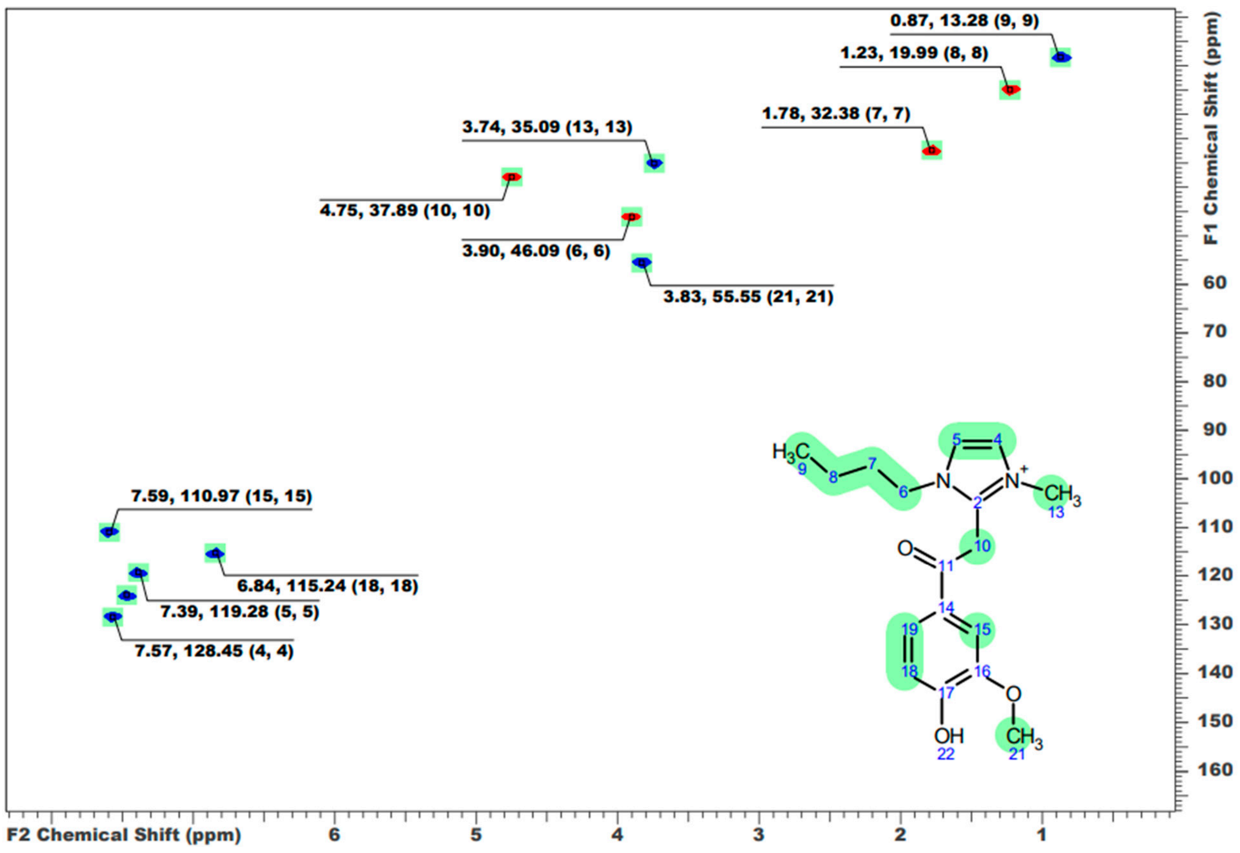

Figure S14. 2D HSQC NMR calculated spectrum and structure of the acetovanillone reaction product 2b.

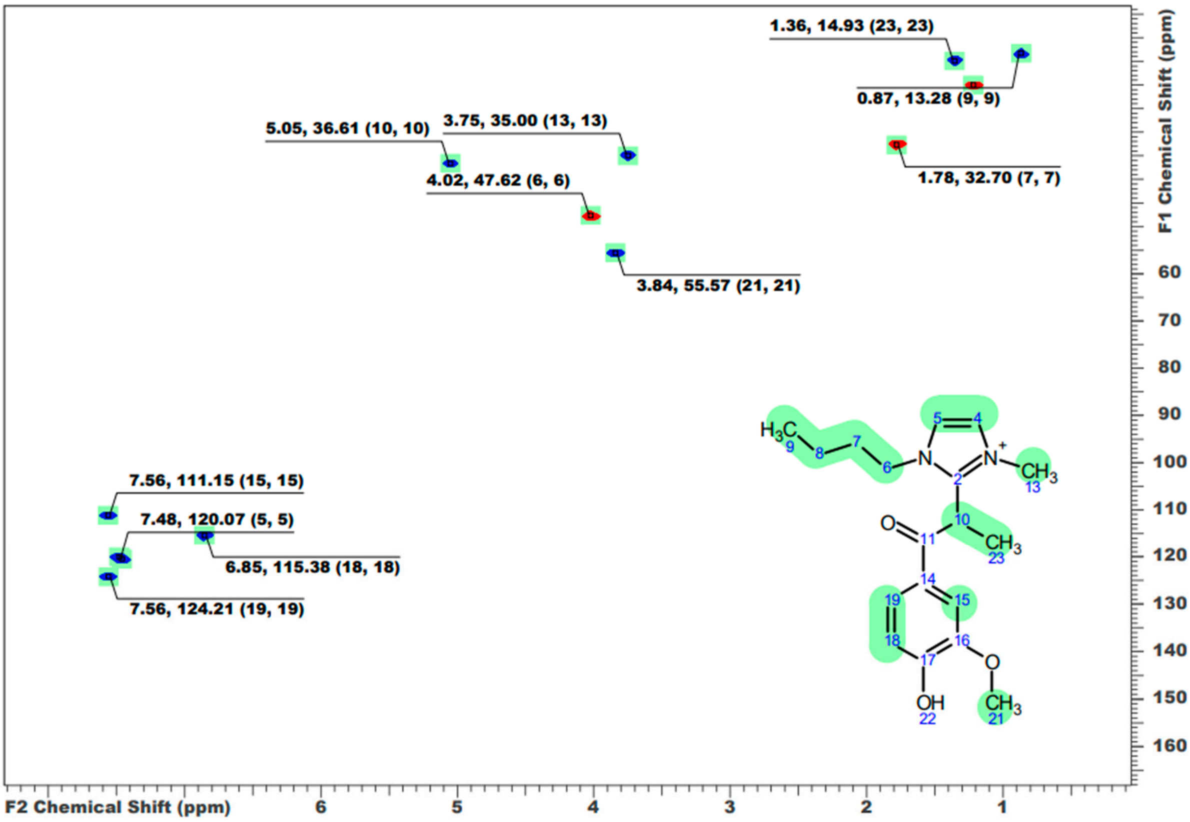

Figure S15. 2D HSQC NMR calculated spectrum and structure of the acetovanillone reaction product 2c.

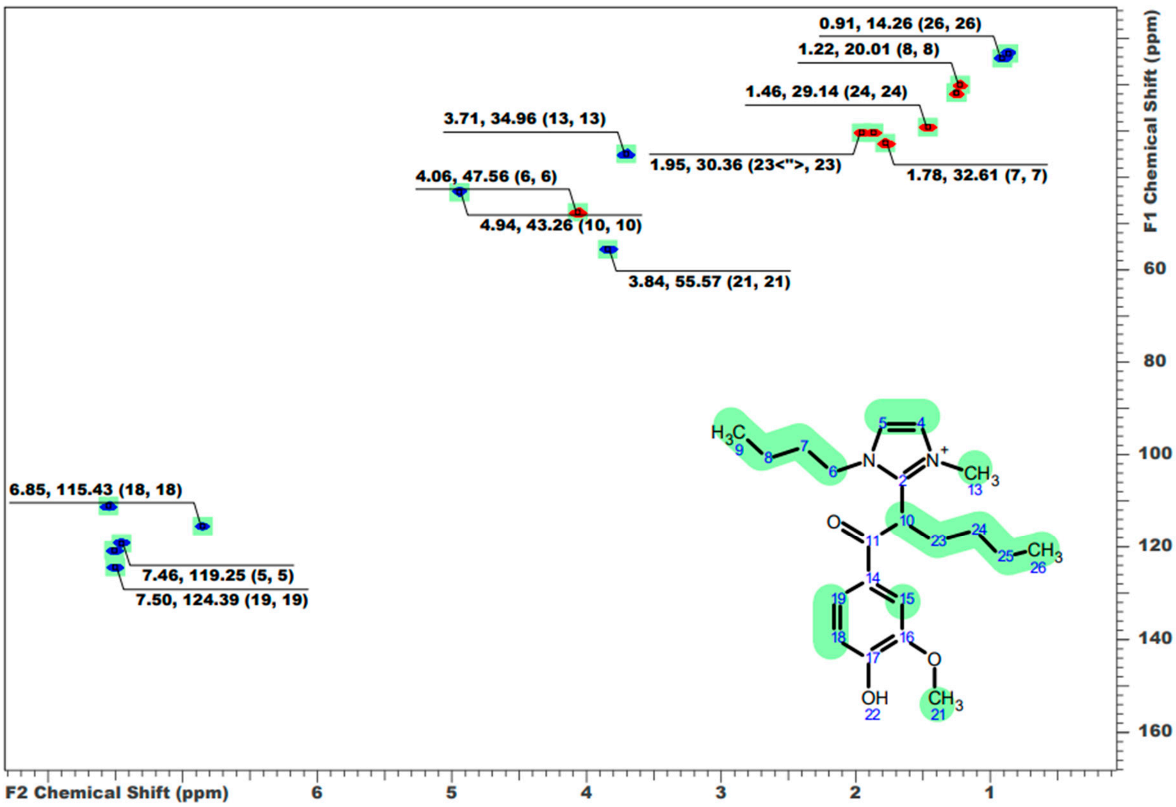

Figure S16. 2D HSQC NMR calculated spectrum and structure of the acetovanillone reaction product 2d.

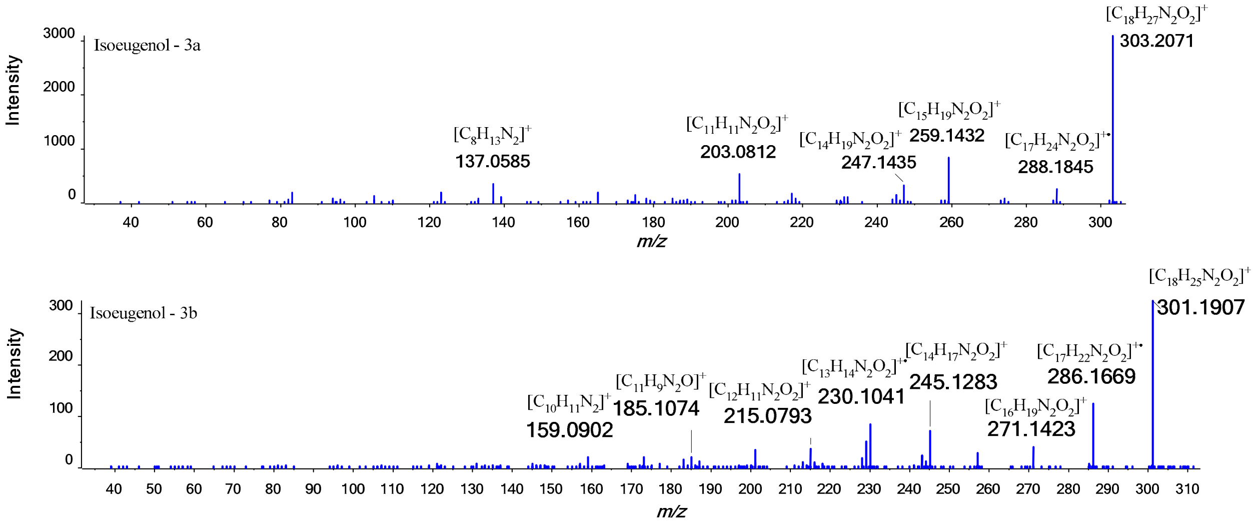

Figure S17. Tandem (CID) mass-spectra of isoeugenol 3a,b reaction products.

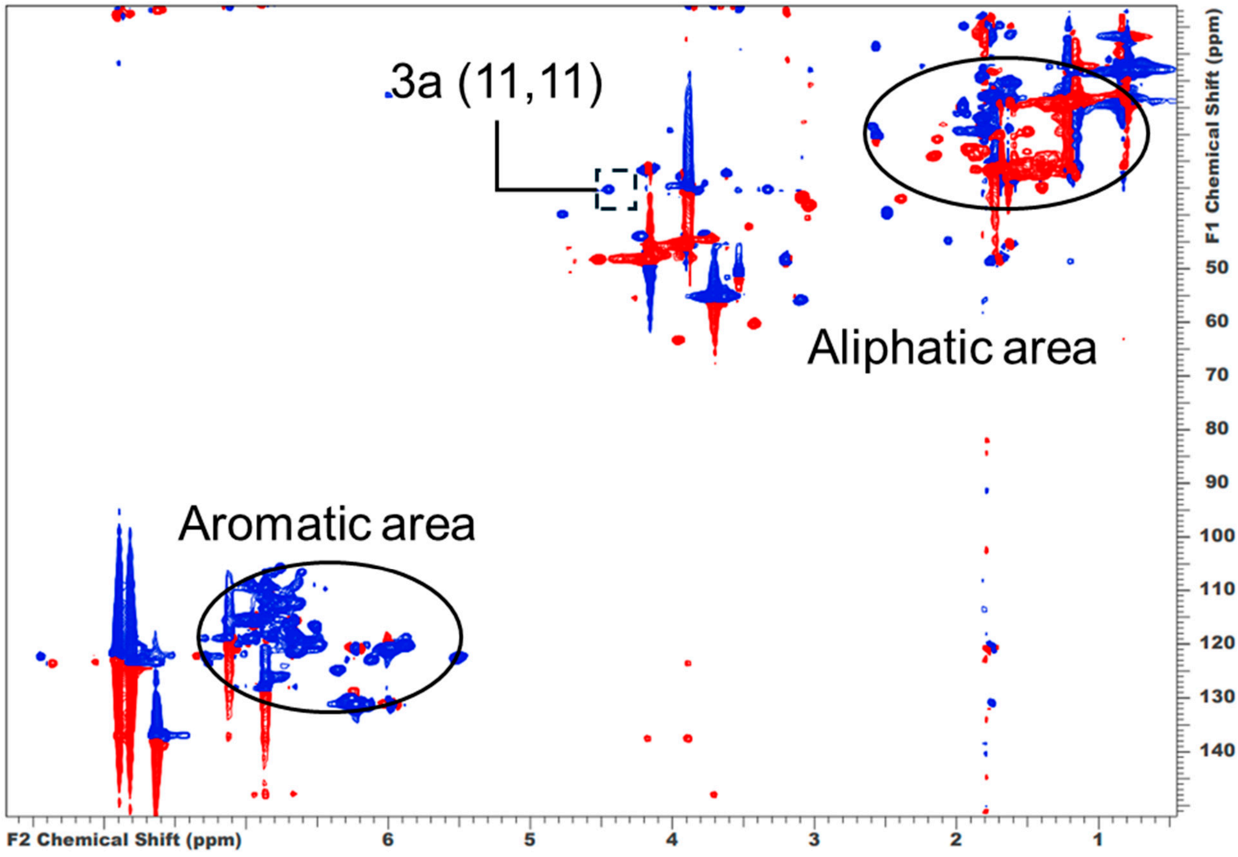

Figure S18. 2D NMR spectrum of the isoeugenol-BmimOAc reaction mixture.

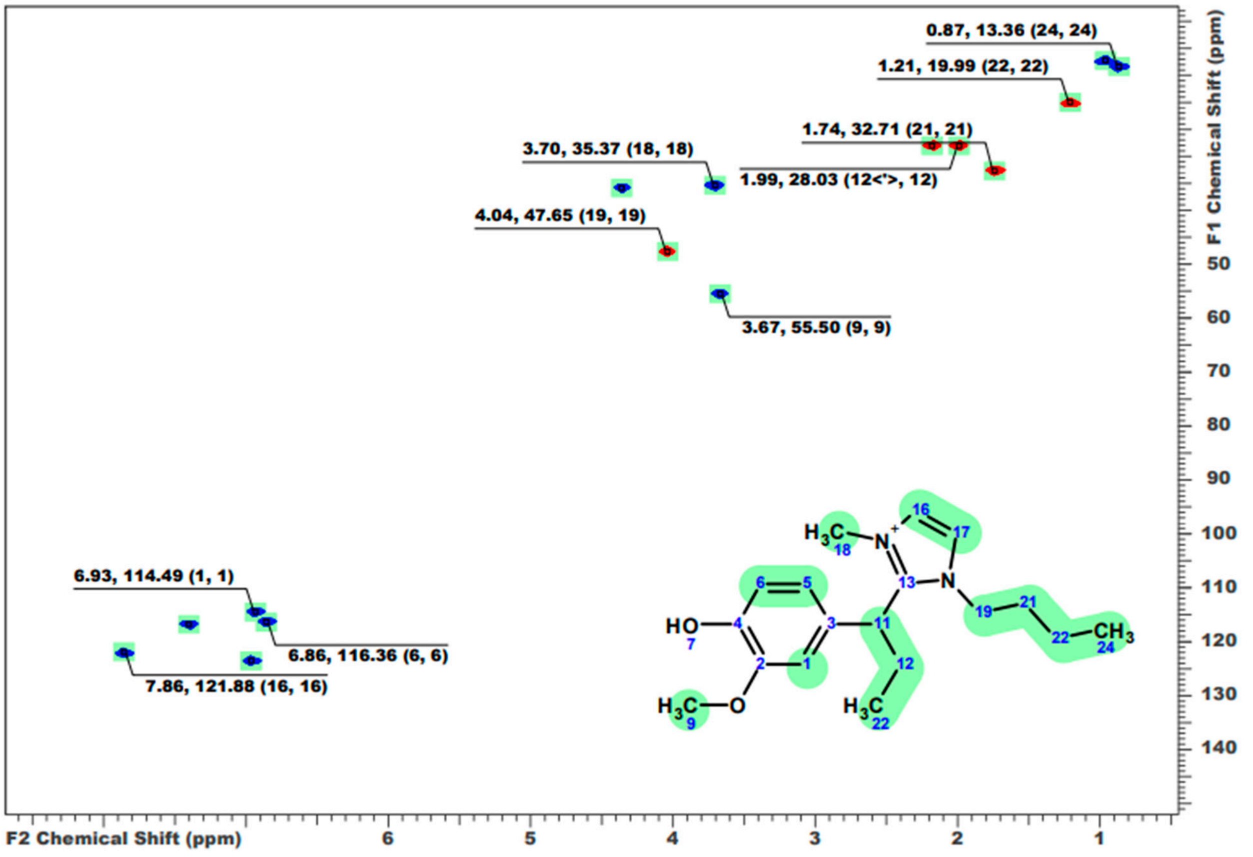

Figure S19. 2D NMR calculated spectrum and structure of the reaction product 3a.

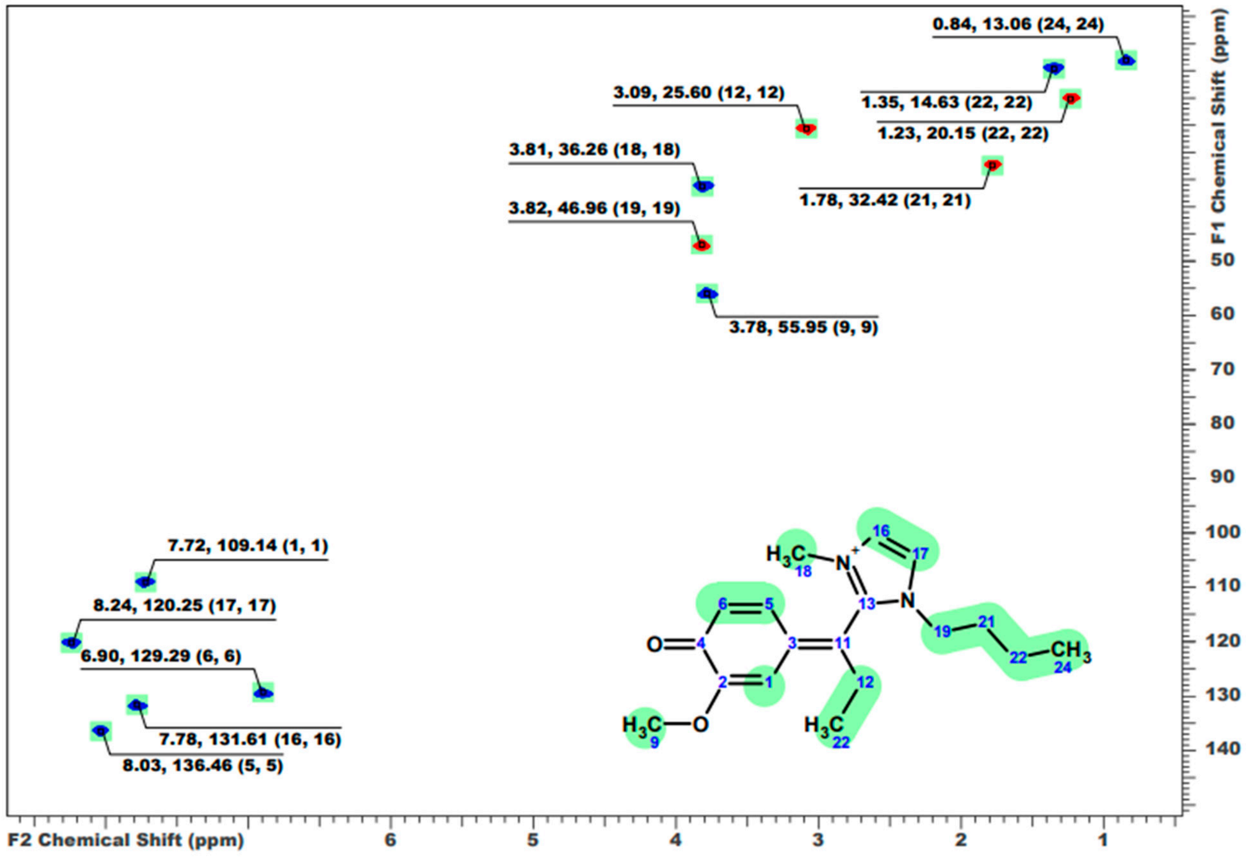

Figure S20. 2D NMR calculated spectrum and structure of the reaction product 3b.
